# Rethinking secondary immunodeficiency: a cross-domain pathway framework for risk stratification

**DOI:** 10.3389/fimmu.2026.1866662

**Published:** 2026-08-26

**Authors:** Iqra Mumtaz

**Affiliations:** Management of Digital Transformation (MDT), IMT School for Advanced Studies Lucca, Lucca, Italy

**Keywords:** hypogammaglobulinemia, secondary immunodeficiency, immunocompromised host, immunoglobulin replacement therapy, infection susceptibility, LMIC, pathway-driven framework, risk stratification

## Abstract

**Background:**

Secondary immunodeficiency (SID) arises from malignancy, immunomodulatory therapies, organ dysfunction, chronic infection, malnutrition, and aging. Despite its increasing prevalence, SID remains conceptualized within disease-specific silos, resulting in inconsistent diagnostic standards and fragmented management across specialties.

**Methods:**

Using the PRISMA 2020 guidelines and a prospectively registered OSF protocol (6qvkx), a systematic, qualitative synthesis was conducted, comprising structured screening, standardized data extraction, and a design-specific risk-of-bias assessment. A search of the MEDLINE, Embase, Scopus, and Web of Science databases resulted in 11,968 records. A Python-assisted fuzzy-matching algorithm identified 9,295 duplicate records (78%), which were confirmed in Zotero. Following deduplication, 2673 unique studies were screened; 100 met the predefined PICOS criteria and were included in the qualitative synthesis. No primary data were collected. The primary reviewer selected the studies and extracted data manually, using Python only for descriptive summarization. Thematic synthesis was presented along three predetermined axes of analysis: *humoral dysfunction, cellular disruption, and innate immune dysregulation.*

**Results:**

Humoral failure, cellular disruption, and innate immune dysregulation were identified as 3 immunologic pathways common across all included clinical domains. A significant difference was noted in the type of diagnostic criteria used: IgG thresholds were used in 78% of the hematological malignancy studies and 42% of the HIV/malnutrition studies, whereas the functional assessment of antibody in the former was employed in 32% of studies compared to 67% of the latter, presumably due to the superior clinical correlation in chronic infection and nutritional deficiency settings. The most common outcome reported was infection burden (range 63-85%). Structured qualitative convergence mapping was used to create an exploratory conceptual risk stratification framework based on variables that met preset cross-domain reproducibility criteria. The framework is designed to provide a structured clinical reasoning tool, organized around convergent cross-domain evidence, which requires prospective validation before use in the clinical setting.

**Conclusion:**

Three immunological pathways converge in the selected SID-inducing conditions: humoral dysfunction, cellular disruption, and innate immune dysfunction. The proposed framework provides a structured approach to risk evaluation and management across specialties and resource settings.

**Systematic Review Registration:**

https://osf.io/6qvkx, identifier 6qvkx.

## 1 Introduction

Secondary immunodeficiency (SID) is a failure of immune competence acquired through underlying disease, treatment, nutritional deficiency, or physiological aging. SID is dynamic, partially reversible, and directly influenced by modifiable exposures, whereas primary immunodeficiencies are constitutional or genetic (1). The burden of SID has increased substantially over the past two decades owing to the widespread adoption of B-cell-depleting biologics, advances in cancer therapeutics, the emergence of cellular immunotherapies, population aging, and expansion of transplant programs (2, 3). Hematology literature has increasingly documented hypogammaglobulinemia associated with targeted therapies, including rituximab and CAR-T cell therapy (4). In recent years, the rheumatology literature has primarily concentrated on monitoring immunoglobulin levels in biologic recipients (5). Immune complications are addressed within specialist disciplines of nephrology and transplant medicine. Conceptualizations of SID have varied across specialties, leading to distinct diagnostic criteria and management strategies, as well as differing laboratory testing methods and inconsistent access to preventive measures such as immunoglobulin replacement therapy (IgRT), vaccines, and antimicrobial prophylaxis (6). This fragmentation carries important clinical consequences, such as delayed diagnosis, non-uniform access to IGRT, and avoidable morbidity due to infection.

This review is founded on the premise that for conditions leading to SID, three pathways of immunological effect can be attributed to the underlying disease: (i) humoral failure, (ii) disruption of cells, and (iii) innate immune dysfunction. This perspective supports reconceptualizing SID as a pathway-driven syndrome.

Several syntheses have investigated single aspects of immune dysfunction, such as antibody deficiency in hematological malignancy (7) and immune reconstitution after transplantation (8). However, these studies have not all included mechanistic data from five SID-producing conditions, have not comprehensively incorporated these data into a three-axis analytic model, and have not developed a cross-specialty clinical translation framework. This review addresses these three important gaps in the evidence base. Existing reviews are structurally limited by: (i) domain-specific scope; (ii) reliance on single-pathway interpretation; and (iii) absence of integrative clinical translation models.

The primary objective of this review is to synthesize evidence on immunological mechanisms, infection phenotypes, diagnostic strategies, and management approaches across five acquired SID domains. Secondary objectives are: (i) to illustrate mechanistic convergence using a consistent, three-axis analytical model; (ii) to propose an exploratory, cross-domain SID Risk Stratification Framework derived from this synthesis; (iii) to examine global applicability with specific attention to low- and middle-income country (LMIC) contexts; and (iv) to identify priority evidence gaps for future investigation.

Importantly, the proposed framework should not be interpreted as a replacement for disease-specific diagnostic guidelines. Rather, it is intended as a cross-specialty conceptual scaffold designed to facilitate recognition of convergent immune dysfunction patterns that may otherwise remain compartmentalized within specialty-specific diagnostic paradigms.

## 2 Materials and methods

### 2.1 Study design and protocol

This study was conducted as a systematic evidence synthesis with qualitative thematic analysis and conceptual framework development, reported in accordance with the Preferred Reporting Items for Systematic Reviews and Meta-Analyses (PRISMA 2020) guidelines (9). Preliminary scoping, limited to reviewing titles of recent narrative reviews to confirm the PICOS question was answerable, was conducted before registration; no database searches were performed during this phase.

The protocol was prospectively registered on the Open Science Framework (OSF; https://osf.io/6qvkx) on February 3, 2026. OSF was selected as the registration platform because this review incorporates qualitative synthesis and conceptual framework development alongside empirical evidence synthesis; PROSPERO is optimized for systematic reviews with quantitative outcomes and does not accommodate the framework derivation component of this study’s design. The OSF registration provides equivalent transparency and pre-specification of eligibility criteria, analytical axes, and synthesis procedures. All eligibility criteria, analytical axes, and extraction procedures were predefined in the registered protocol and remained unaltered throughout the review.

No automated screening algorithms or machine-learning tools were employed at any stage of study selection, eligibility assessment, or data extraction. Screening and eligibility decisions were conducted manually by the primary reviewer using predefined protocol-based criteria and standardized extraction templates. All findings were derived from previously published studies. Computational tools used for deduplication and post-extraction data management are described in §2.4.

The research question was structured using the PICOS framework: *Population:* adults and children with secondary immunodeficiency (SID) due to acquired conditions; *Intervention/Exposure:* underlying diseases, immunosuppressive therapies, or other SID-associated exposures; *Comparator:* patients without SID or differing exposure groups where applicable; *Outcomes:* immunological abnormalities, infection burden, diagnostic approaches, and management strategies; *Study design:* randomized and non-randomized studies with extractable empirical data.

This review is a qualitative systematic evidence synthesis designed to uncover recurring patterns of convergence, both mechanistic and clinical, across the SID domains. The resulting framework, conceptual and exploratory, aims to structure convergent evidence signals into a clinically interpretable structure rather than to generate or test causal or predictive hypotheses. Hypothesis testing is methodologically precluded by the qualitative synthesis design, the heterogeneity of included study types, and the absence of statistical pooling; the appropriate role of this review is to synthesize pre-existing evidence into a structured format that can inform and motivate future prospective hypothesis-driven investigation.

### 2.2 Eligibility criteria

Studies were eligible if they included patients with confirmed and/or clinically inferred SID from acquired conditions (such as hematological malignancy, biologic or targeted immunotherapy, autoimmune or inflammatory disease, solid organ or HSCT, chronic organ dysfunction [CKD or hepatic failure], or chronic infections [HIV or SAM] or physiological aging (10).

Eligible study designs included randomized controlled trials, observational studies, registry analyses, and systematic reviews with extractable empirical data. Studies were eligible only if published between January 2005 and March 2026, a restriction selected to reflect the contemporary therapeutic landscape of B-cell-depleting biologics, targeted immunotherapies, and cellular therapies.

Additionally, a limited number of high-impact conceptual, policy, and methodological papers were included to inform framework development where empirical evidence was insufficient. Inclusion required reporting at least one relevant outcome: infection incidence or severity (11), immunoglobulin concentrations (12), vaccine immunogenicity (13), or lymphocyte subset data (14).

A qualitative synthesis approach was used due to substantial heterogeneity. The final corpus of 100 included studies represents a deliberately scoped, high-specificity dataset. The scope was defined to capture studies addressing acquired SID across five predefined clinical domains within the contemporary therapeutic era (January 2005 to March 2026), rather than to achieve exhaustive coverage of all immune dysfunction literature. In secondary immunodeficiency research, where mechanistic heterogeneity across disease domains is high and quantitative pooling is methodologically precluded, a corpus of 100 studies with extractable empirical data across five domains constitutes a rigorous and sufficient evidence base for qualitative convergence mapping and structured framework development.

Exclusion criteria comprised studies focused exclusively on primary immunodeficiency, non-human studies, case reports or small case series without generalizable data, conference abstracts without full-text availability, studies lacking extractable outcomes, and non-English-language publications, the latter because translation resources were unavailable. Systematic reviews were included only where their constituent primary studies were not independently retrievable, or where they provided conceptual and policy-level information not otherwise captured in primary empirical studies. All systematic reviews were additionally assessed using AMSTAR-2 during the eligibility screening stage; those rated as low or critically low quality were excluded from the final corpus and are not represented among the 20 retained systematic reviews in the 100 included studies.

### 2.3 Information sources and search strategy

A literature search was performed across four electronic databases: MEDLINE (PubMed), Embase, Scopus, and Web of Science, using controlled vocabulary (MeSH/Emtree terms) and free-text keywords. The main search terms were: *secondary immunodeficiency, hypogammaglobulinemia, immune dysfunction, susceptibility to infection, immunosuppressive therapy, transplantation, chronic kidney disease, HIV, malnutrition, and immunosenescence.* A manual backward-reference and forward-citation search of key included studies was also performed. The complete search strategy, including database-specific Boolean strings and the script used for automated PubMed retrieval, is provided in the **Supplementary Materials**.

### 2.4 Study selection and data extraction

Search results from all four databases were exported to Zotero, where an initial computational duplicate-flagging pass (based on title similarity and DOI matching) identified candidate duplicates; all flagged candidates were manually confirmed and removed, yielding 9,295 duplicates removed (78%) from 11,968 to 2,673 unique records. All study selection, eligibility screening, and data extraction were performed manually by the primary reviewer using the standardized, protocol-specified extraction template.

Computational tools were used only for two administrative functions with no analytical or interpretive role: (i) the duplicate-flagging step described above, and (ii) post-extraction tabulation of descriptive statistics from data already extracted manually. No automated or machine-learning tools were used for screening, eligibility assessment, thematic synthesis, or framework derivation. Full details of the search execution, deduplication, and data-management scripts are provided in the **Supplementary Materials**.

Although the registered protocol specified dual independent review, this was not feasible due to resource constraints inherent to a single-author PhD project, and is acknowledged as the principal methodological limitation of this review (§4.6). To partially mitigate this, all eligibility criteria were prospectively registered in OSF before database searching; all analytical axes and extraction procedures were predefined and remained unchanged throughout the review; screening followed a structured two-stage process using standardized written eligibility criteria (**Supplementary Appendix A**); and standardized extraction templates were used throughout. Internal consistency was assessed by the primary reviewer through re-evaluation of a random subsample of 200 records (Cohen’s κ = 0.80), as detailed in §4.6. Every inclusion/exclusion decision and its rationale are recorded in **Supplementary Table 1**, enabling *post-hoc* auditability.

All scripts used for search execution, deduplication, and post-extraction summarization are provided in full as annotated code files in the **Supplementary Materials** and are additionally available via the OSF repository (https://osf.io/6qvkx). These tools performed no analytical, interpretive, or synthesis functions in this review.

### 2.5 Risk of bias assessment

This study assessed the risk of bias in all randomized trials using the Cochrane Risk of Bias 2 (RoB2) tool, and in all observational studies and registry-based analyses using the Newcastle-Ottawa Scale (NOS) (NOS ≥7 was set as the criterion for high quality). AMSTAR-2 was applied to all candidate systematic reviews during eligibility screening (§2.2); only systematic reviews rated as moderate or high quality were retained in the final corpus, and the 20 systematic reviews included in this review reflect this filtered set. Registry-based studies (n = 12) were evaluated using an adapted NOS, in line with published instructions for its use in registry-based studies. Among the included randomized trials (n = 20), 11 (55%) were rated low risk, 7 (35%) as some concerns, and 2 (10%) as high risk. The 48 observational studies included 31 (65%) that were of high quality. Of the 12 studies that were based on the registry, 8 (67%) were scored as high-quality (NOS ≥7), 3 (25%) were scored as moderate-quality (NOS 5–6), and 1 (8%) was scored as low-quality (NOS <5); the latter was interpreted with caution and was identified explicitly where it was used in the synthesis.

### 2.6 Synthesis method

There was substantial methodological and clinical heterogeneity in how SID was defined, the outcome measures used, and the study design — heterogeneity that is not incidental but is the central object of study of this review. The five included domains (hematological malignancy/CAR-T, autoimmune disease on biologics, transplantation/CKD, HIV/malnutrition, and immunosenescence) were deliberately selected because they represent maximally divergent clinical contexts that nonetheless share a common downstream phenotype: *acquired immune dysfunction with elevated infection risk* (15). This heterogeneity precludes quantitative pooling, and a qualitative synthesis was therefore selected as the only methodologically appropriate approach.

Critically, the unit of analysis in this review is not ‘the patient’ or ‘the disease’ but the immunological pathway (humoral, cellular, innate) — a level of abstraction at which convergence across disparate clinical contexts is both biologically plausible and empirically supported, even though the upstream disease processes (e.g., CAR-T-induced B-cell aplasia versus malnutrition-induced global immune suppression) remain pathophysiological distinct and require domain-specific management (16). The synthesis was carried out in three stages: (i) line-by-line coding of extracted findings within each domain; (ii) axial aggregation of domain-level themes onto the three predefined pathway axes; and (iii) cross-domain comparison of convergence and divergence in mechanisms, diagnosis, and management at the pathway level only — not at the level of disease-specific treatment recommendations.

Studies were grouped into predefined clinical domains, and extracted findings were mapped onto the three predefined analytical axes. No statistical pooling or meta-analytic modeling was performed (16). Evidence certainty was appraised qualitatively, guided by the four GRADE domains: risk of bias, consistency of findings, directness to the review question, and completeness of outcome reporting, without generating formal GRADE summary-of-findings tables. Formal GRADE tables were not produced because the qualitative synthesis design and the heterogeneity of outcome measures across domains precluded the domain-level certainty ratings that GRADE summary tables require. Instead, narrative certainty appraisals are integrated into each domain subsection of the Results and are summarized in the Discussion.

### 2.7 Framework development methodology

The SID Risk Stratification Framework was developed through a structured, convergence-based approach grounded in the qualitative synthesis of included studies. Framework variables were identified through a systematic evaluation of extracted data across all clinical domains.

A variable was eligible for inclusion if it satisfied two predefined criteria: (i) it was reported in ≥10% of included studies (i.e., ≥10 of 100 studies) or identified across ≥3 independent clinical domains; and (ii) it showed a consistent association with clinically meaningful outcomes (14). Variables meeting the frequency or domain threshold but lacking consistent association with the outcome were retained as contextual or supportive variables only, as detailed in **Supplementary Table 2**. This dual-threshold approach ensured that variable selection reflected both reporting frequency and cross-domain reproducibility.

Variables with these properties were classified according to the three axes of analysis (humoral, cellular, innate), immune function, clinical history, therapeutic exposure, and host factors (15). To minimize subjectivity, a set of weightings was defined and applied to each variable, based on reproducibility, clinical relevance, and across-domain frequency (16).

The resulting framework represents a structured conceptual synthesis model designed to organize reproducible cross-domain evidence into a clinically interpretable format to support structured clinical reasoning and research prioritization. There is no evidence of discrimination testing (e.g., AUC/C-statistic), calibration assessment, or external validation against patient-level outcomes, the three minimum standards for any clinical risk score before bedside use, as set out in the TRIPOD reporting guideline for prediction model studies (17, 18). Clinicians are advised to interpret the numerical values in **Table 1A** as a transparent record of evidentiary convergence rather than as validated weights that denote actual marginal contributions to infection risk.

**Table 1A:** Proposed SID risk stratification framework: variables, criteria, and scoring.

Domain	Variable	Criteria	Points	Rationale
Humoral	*IgG <4 g/L*	Severe hypogammaglobulinemia	2	Consistently associated with impaired opsonization and infection risk (1, 11, 19, 20)
Humoral	*IgG 4–6 g/L + poor vaccine response*	Functional antibody failure despite borderline total IgG	2	Functional antibody failure predicts infection risk better than IgG alone (17–19)
Exposure	*B-cell depleting or intensive therapy*	Anti-CD20, anti-CD38, CAR-T, BTK inhibitor, or HSCT conditioning: current or within 12 months	2	Disrupts B-cell maturation and antibody production (2, 6, 13, 17)
Clinical	*≥2 severe infections per year*	Requiring hospitalization or intravenous antimicrobials	3	Strongest indicator of clinically relevant immune dysfunction (7, 19, 21)
Host factor	*High-risk underlying condition*	CKD stage 4–5 (eGFR <30 mL/min/1.73 m²); HIV with CD4 <200 cells/µL; solid organ transplant within 24 months; or SAM	1	Associated with systemic immune dysregulation (22–24)

**Table 1B:** Risk band classification.

Risk bands			
Score	Risk level	Clinical interpretation	Recommended actions
0–2	Low	Immune reserve preserved	annual monitoring + vaccination
3–5	Moderate	Early functional immune impairment	monthly monitoring + functional antibody testing
6–10	High	Clinically significant secondary immunodeficiency	specialist review + consider IgRT + targeted antimicrobial prophylaxis

The SID Risk Stratification Framework should not be interpreted as a clinical prediction rule, diagnostic instrument, treatment algorithm, or validated prognostic score. This framework is a research prioritization tool, not a clinical decision-support instrument, and must not be used to guide individual patient management. It is an exploratory model derived from qualitative thematic synthesis of 100 included studies. Variable inclusion and point values reflect reporting frequency and cross-domain reproducibility in the existing literature — they do not represent validated effect sizes, odds ratios, or hazard ratios for infection risk. No discrimination (AUC), calibration, or external validation has been performed. Prospective validation against patient-level infection outcomes, following TRIPOD methodology, in defined cohorts across disease domains, age groups, and health system contexts, is a prerequisite for any clinical application of this framework. It is proposed as the primary next research step in §4.6. Numbers in parentheses refer to the citation cluster supporting each criterion. BTK, Bruton tyrosine kinase; CAR-T, chimeric antigen receptor T cell; CKD, chronic kidney disease; eGFR, estimated glomerular filtration rate; HSCT, hematopoietic stem cell transplantation; IgRT, immunoglobulin replacement therapy; SAM, severe acute malnutrition.

A complete derivation of the logic, variable scoring criteria, risk band classification thresholds, sensitivity analysis results, and the resulting framework tables is provided in Section 3.6, including an explicit roadmap for the prospective validation study required before any clinical use. Because outcome definitions, study populations, follow-up durations, and SID-producing conditions differed substantially across included studies, no common effect measure existed that would permit meaningful pooled estimation or derivation of evidence-based coefficients; this is the methodological basis for the reporting-frequency weighting approach described above.

## 3 Results

### 3.1 Study selection

**Figure 1:**
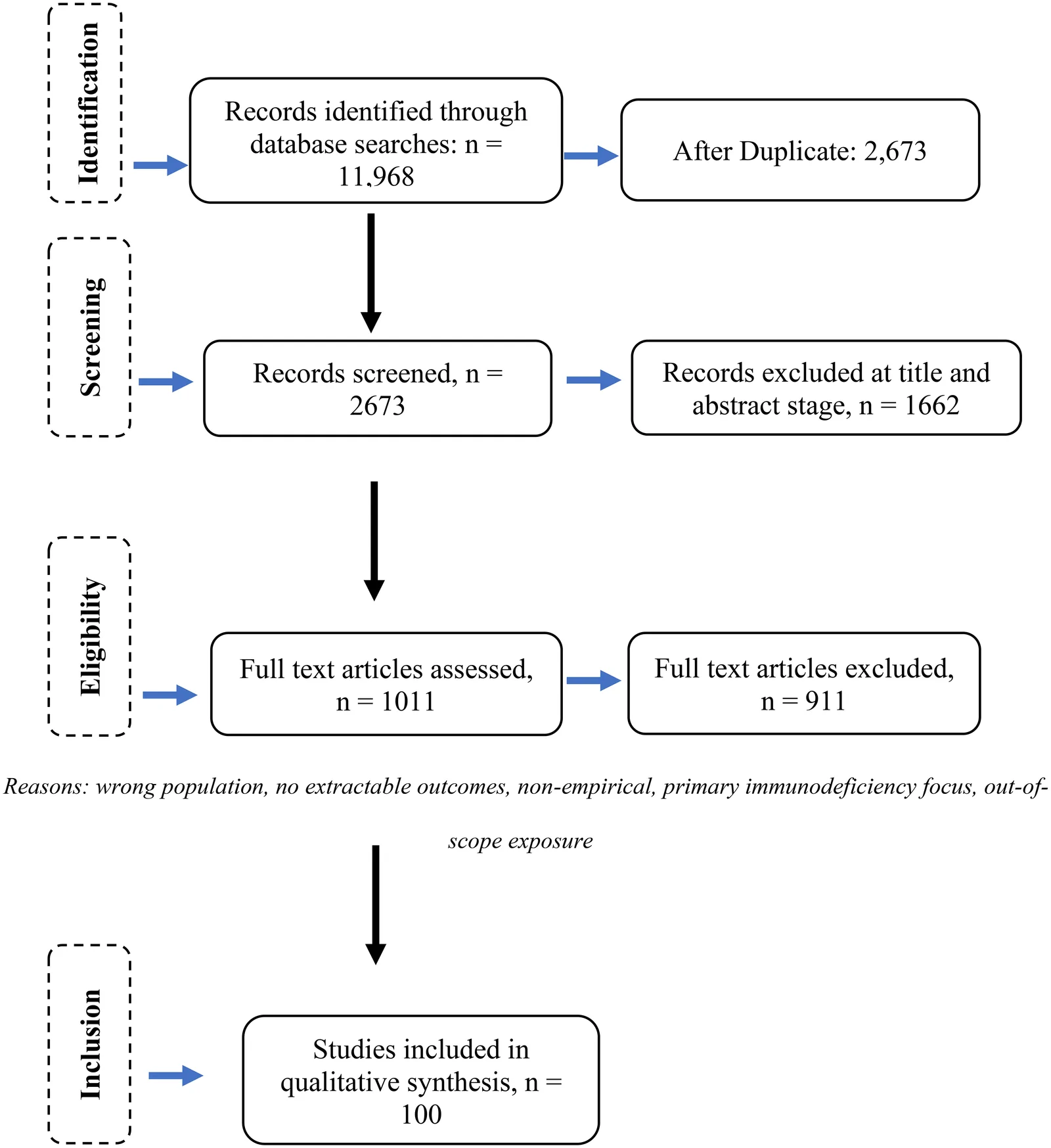
Flow chart for PRISMA approach. Four-stage PRISMA flow illustrating identification (n = 11,968), screening after deduplication (n = 2,673), full-text assessment (n = 1,011), and final inclusion (n = 100). Numbers at each stage are reconciled with the study selection narrative in Section 3.1. Reasons for full-text exclusion are shown within the diagram. This structure aligns with PRISMA 2020 reporting standards and supports reproducibility of the study selection process.

Database searches identified 11,968 records. Removal of duplicate data (n = 9,295) left 2,673 unique records for screening. Of the 2,673 unique records, 1,662 were excluded at the title and abstract screening stage as clearly incompatible with the predefined PICOS criteria based on the population or exposure. Full texts were subsequently obtained for the remaining 1,011; 911 were discarded after full-text review (most of which had a primary immunodeficiency focus only; were non-empirical (e.g., editorials, commentaries with no extractable data); or had no reportable immunological or infection outcomes). A total of 100 studies were included in the qualitative synthesis (**Figure 1**).

### 3.2 Characteristics of the evidence base

The included studies comprised a heterogeneous evidence base: randomized controlled trials (n = 20), observational studies (n = 48), registry-based analyses (n = 12), and systematic reviews (n = 20). The inclusion of systematic reviews (SRs; 20%) warrants methodological clarification. Each included SR was evaluated against the full list of primary studies in the corpus; where an SR’s constituent primary studies were independently identified and included, SR-level data were used solely for framework-level and policy insights rather than primary mechanistic synthesis, to minimize evidence overlap. SR findings are identified by study type (SR) throughout the text and in **Supplementary Table 1**, enabling readers to assess the independence of the evidence base.

Methodological and guideline-based studies were also included to support risk-of-bias assessment and framework development. Studies from underrepresented regions contributed disproportionately to cohorts examining HIV-related immune dysfunction, severe acute malnutrition, and chronic kidney disease–associated immune impairment (25).

Clinical domains were categorized according to the quantitative mapping presented in **Table 2**. The largest contribution derived from hematological malignancies and treatment-related SID (n = 38), followed by immunosenescence and ageing-related immune dysfunction (n = 20), HIV and malnutrition-associated immune impairment (n = 19), autoimmune and inflammatory diseases receiving biologic or targeted therapies (n = 12), and transplantation, chronic kidney disease, and organ failure–associated SID (n = 11) (13). Domain classification was derived from the study-level dataset (**Supplementary Table 1**), with each study assigned to a single primary clinical domain to ensure mutually exclusive categorization.

**Table 2:** Diagnostic heterogeneity and reporting practices across secondary immunodeficiency clinical domains: quantitative mapping of 100 included studies.

Domain	Studies, n	IgG threshold use (%)	Functional antibody assessment (%)	Infection reporting (%)	Lymphocyte subset reporting
Hematological malignancies	38	78	32	85	42%
Autoimmune/biologic therapies	12	64	48	72	35%
Transplantation (SOT and HSCT)/CKD and organ failure	11	59	51	69	61%
HIV/malnutrition	19	42	67	81	47%
Aging/Immunosenescence	20	55	46	63	28%

Transplantation (SOT n=5; HSCT n=6) was analyzed as a single domain due to limited study numbers, although these groups differ mechanistically and warrant separate validation in future work. CKD and organ failure were grouped with transplantation because they share overlapping features of secondary immunodeficiency, including chronic immune dysregulation and infection susceptibility. HIV and malnutrition were combined, given their common impact on systemic immune impairment despite distinct etiologies; studies examining aging as a coexisting exposure in these populations were classified within this domain, where immune dysfunction was primarily attributed to infection or nutritional status rather than physiological senescence. Lymphocyte subset reporting (CD4, CD8, CD19, NK) was included as a supportive variable. Functional antibody assessment varied by study and included vaccine-specific titers, opsonic assays, and lymphoproliferative responses. Values reflect reporting practices, not prevalence, and were rounded. IgG thresholds in immunosenescence likely overestimate diagnostic utility due to a lack of age-specific validation.

SID definitions were heterogeneous across studies. The majority relied on immunoglobulin G (IgG) thresholds as primary diagnostic criteria. In contrast, functional antibody assessment was more frequently incorporated into studies of chronic infection and organ dysfunction, where total immunoglobulin levels showed limited correlation with clinical outcomes (26). As summarized in **Table 2**, substantial variability was observed in both diagnostic approaches and reporting practices across domains, particularly in the relative use of IgG thresholds and functional antibody assessment.

### 3.3 Domain-level findings

**Figure 2:**
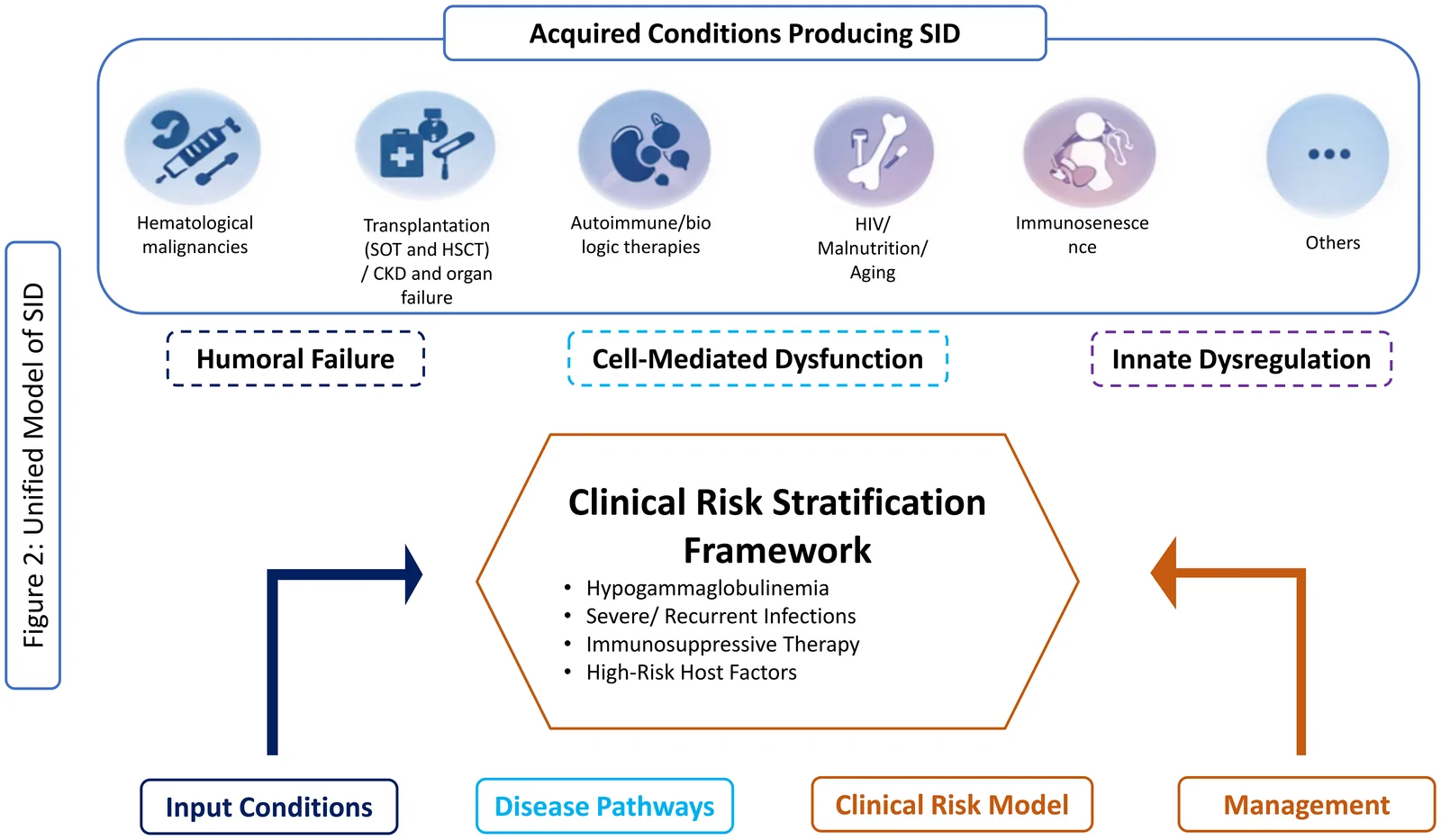
Unified pathway model of secondary immunodeficiency. Conceptual diagram mapping acquired disease domains onto the core immune disruption pathways as summarized in **Table 3**. Arrows indicate the direction from disease exposure through pathway activation to the downstream clinical outcome. Overlapping pathway boundaries reflect the coexistence of multiple mechanisms in individual patients. The right panel summarizes the clinical correlates and primary management implications for each pathway. Management recommendations are illustrative and require adaptation to the individual clinical context. ART, antiretroviral therapy; BTK, Bruton tyrosine kinase; CAR-T, chimeric antigen receptor T cell; CKD, chronic kidney disease; CLL, chronic lymphocytic leukemia; HSCT, hematopoietic stem cell transplantation; IgRT, immunoglobulin replacement therapy; JAK, Janus kinase; SAM, severe acute malnutrition.

Domain-level findings are organized into the five SID categories acquired under the eligibility criteria. The mechanistic mapping of each domain onto the three analytical axes (humoral, cellular, innate) is summarized in **Figure 2**; **Table 3**; domain-level diagnostic and reporting patterns are presented in **Table 2**.

**Table 3:** Cross-domain mechanistic convergence in secondary immunodeficiency.

Pathway	Mechanism	Representative conditions and exposures	Clinical consequence
Humoral dysfunction (1, 13, 20)	*B-cell depletion; polyclonal immunoparesis; impaired Ig class switching*	CLL; multiple myeloma; rituximab/anti-CD20; CAR-T; severe acute malnutrition	Recurrent bacterial infections; poor vaccine seroconversion; susceptibility to encapsulated organisms
Cellular disruption (22, 27, 28)	*CD4 T-cell depletion; naïve T-cell contraction; lymphocyte exhaustion; delayed thymic reconstitution*	HIV; post-HSCT; immunosenescence; CAR-T conditioning; JAK inhibitor therapy	Viral reactivation (CMV, VZV, HSV); opportunistic infections; impaired intracellular pathogen clearance
Innate immune dysregulation (23, 24)	*Complement reduction; impaired phagocyte function; uremic toxin-driven antigen presentation failure*	CKD stage 4–5; hepatic failure; malnutrition; advanced ageing (inflammageing)	Invasive bacterial disease; attenuated acute phase response; impaired vaccine response

CMV, cytomegalovirus; CKD, chronic kidney disease; CLL, chronic lymphocytic leukemia; HSCT, hematopoietic stem cell transplantation; HSV, herpes simplex virus; JAK, Janus kinase; VZV, varicella-zoster virus. This table was developed by the primary reviewer through structured qualitative synthesis of extracted data, applying the predefined variable-eligibility criteria described in §2.7. It was not generated by automated analysis. This table is referenced in **Figure 2**.

#### 3.3.1 Hematological malignancy and treatment-related SID

SID in hematological malignancies reflects concurrent disease biology and treatment effects (15). Baseline hypogammaglobulinemia is present in approximately 85% of CLL patients at diagnosis and worsens progressively with disease stage and treatment lines (29). There is polyclonal immunoparesis in multiple myeloma, even in remission. Modern targeted treatment exacerbates these impairments: BTK inhibitors impair B-cell signaling and the ability to switch classes within the IgG, and serum IgG concentrations fall below 4 g/L in ≥30% of patients receiving long-term ibrutinib therapy (16). Anti-CD38 and bispecific T-cell-engaging antibodies cause severe, sustained hypogammaglobulinemia; new evidence suggests that prophylactic IgRT can be initiated in this setting without regard to absolute IgG levels (30). CAR-T therapy results in prolonged humoral immunodeficiency, with B-cell aplasia persisting beyond 12 months in a substantial proportion of patients and grade 3–4 infections occurring in 20–35% of patients within the first year of follow-up (31). Vaccine responsiveness is markedly attenuated: seroconversion rates to COVID-19 vaccines were suboptimal in patients receiving anti-CD20, anti-CD38, and BTK inhibitor therapies. IgRT shows infection benefit in selected patients, but initiation thresholds and treatment duration vary substantially between centers, reflecting the absence of cross-specialty consensus (32). As illustrated in **Table 4**, quantitative immunoglobulin thresholds exhibited limited correlation with clinical infection outcomes in this domain.

**Table 4:** Cross-domain synthesis of immunological dysfunction, infection patterns, and clinical implications in secondary immunodeficiency.

Domain	Immunological mechanism	Key findings	Infection pattern	Diagnostic limitations	Management implications
Hematological malignancy (1–4, 11, 13)	Humoral dysfunction; B-cell depletion	Severe hypogammaglobulinemia common	Recurrent bacterial infections	IgG thresholds are poorly predictive	IgRT is beneficial but inconsistently applied
Autoimmune disease (biologics) (8, 17, 31, 33)	Treatment-induced antibody deficiency	Anti-CD20 therapy reduces Ig levels	Opportunistic and viral infections	Baseline variability is not always considered	Monitoring + selective IgRT
Transplantation (34–36)	Immune suppression + delayed reconstitution	Combined immune deficits	Early high-risk infections	Lack of standardized timing for testing	Prophylaxis + revaccination
CKD/organ failure (23, 24)	Innate + adaptive dysfunction	Complement and antigen presentation defects	Pneumonia, bacteremia	Rarely assessed clinically	Limited targeted management
HIV/chronic infection (22, 27, 37)	Cellular immune disruption	Persistent immune activation	Opportunistic infections	IgG alone insufficient	ART + immune monitoring
Malnutrition (33, 38)	Global immune suppression	Reduced antibody and cellular function	Severe recurrent infections	Minimal diagnostic capacity	Nutrition + infection prevention
Aging (28, 39)	Immunosenescence	Decline in T-cell and B-cell function	Respiratory infections	No age-adjusted thresholds	Vaccination + monitoring

IgG, immunoglobulin G; IgRT, immunoglobulin replacement therapy; ART, antiretroviral therapy; CKD, chronic kidney disease.

#### 3.3.2 Autoimmune and inflammatory disease on biologics or targeted agents

In autoimmune conditions, SID is predominantly treatment-mediated. Rituximab and other anti-CD20 therapies cause clinically significant secondary antibody deficiency in 10–30% of long-term recipients; IgG levels may fall to levels indistinguishable from those seen in common variable immunodeficiency (21). Pre-treatment immunoglobulin levels predict post-rituximab hypogammaglobulinemia, supporting baseline serological assessment before initiating B-cell-depleting therapy (23). Anti-TNF agents increase susceptibility to bacterial and granulomatous infections; JAK inhibitors disrupt cytokine signaling relevant to antiviral defense, producing increased herpes zoster rates and attenuated vaccine responses (30). Infection risk is directly related to cumulative immunosuppressive dose, and glucocorticoids independently contribute to infection susceptibility, particularly in combination regimens. In long-term autoimmune cohorts, however, functional antibody assessment is more discriminating than IgG measurements alone (36). **Table 4** summarizes these domain-specific patterns alongside their diagnostic implications.

#### 3.3.3 Transplantation, CKD, and organ failure

In transplantation, complex SID arises from interactions among conditioning, allograft immunosuppression, graft-versus-host phenomena, and incomplete immune reconstitution. Concurrent immunosuppression, graft-related factors, and delayed immune reconstitution put patients at the highest risk of infection during the early post-transplant period (40). In this setting, immune suppression is wide-ranging, depending on the intensity and type of conditioning and the host’s underlying immune status, thus requiring careful risk stratification. There is no consensus on the optimal time for revaccination after HSCT, and there is a significant inter-center variability in the timing of initiating IgRT after HSCT (41). Interestingly, dysregulated complement activation, diminished antigen presentation, lymphocyte exhaustion, and diminished vaccine responses are all immune dysfunctions that are under-recognized and are associated with CKD (42).

Advanced CKD patients experience elevated rates of pneumonia and bacteremia, as well as decreased antibody responses to influenza and pneumococcal vaccines. Nevertheless, comprehensive immunological assessment is not routinely incorporated into nephrology practice (43). Since hepatic failure impairs the ability to opsonize pathogens, its reduced production of complement proteins and the dysfunction of Kupffer cells increase the risk of infection. Results suggest that, despite this, only 51% of studies on transplantation and CKD used antibody function as a diagnostic test, underscoring the importance of standardizing diagnostic methods using functional antibodies.

#### 3.3.4 HIV, malnutrition, and coinfection-associated immune dysfunction

Even with effective antiretroviral therapy, HIV is associated with persistent immune dysfunction, including CD4 impairment, chronic immune activation, and altered B-cell memory (44). The effects in LMICs are exacerbated because individuals acquire tuberculosis, endemic fungi, and parasite infections, which may mimic advanced primary immunodeficiency. Thymic involution, lymphocyte functional impairment, decreased immunoglobulin production, and barrier disruption occur in children who experience severe acute malnutrition (45). Nutritional rehabilitation leads to immune system restoration in people who have high-burden conditions, but this process results in partial recovery when they have both HIV and enteric infection (46). As shown in **Table 2**, studies in this domain more frequently applied functional immune assessment methods consistent with evidence that functional indices correlate more reliably with clinical outcomes than total immunoglobulin quantification in populations with chronic infection and nutritional deficiency.

#### 3.3.5 Immunosenescence and age-related vulnerability

Aging produces progressive immune remodeling termed immunosenescence, characterized by thymic involution, contraction of naïve T-cell pools, declining B-cell class switching, and dysregulated innate immune signaling manifest as chronic low-grade inflammation (inflammageing) (47). The changes lead to decreased vaccine effectiveness, worsened respiratory infections, and longer recovery times after infection in patients receiving immunosuppressive treatments (48). A consistent limitation across included studies was the absence of age-specific SID diagnostic thresholds, although current criteria were based on younger study populations. Application of diagnostic thresholds derived from younger study populations to elderly, multimorbid, or treatment-exposed patients risks systematic underestimation of clinically meaningful immune dysfunction (49). **Table 4** shows that the absence of age-specific reference ranges creates a particular diagnostic restriction in this area.

### 3.4 Cross-domain quantitative mapping

**Table 2** presents a descriptive quantitative mapping of diagnostic and reporting practices across clinical domains, derived from study-level data extraction (**Supplementary Table 1**). Two interpretive caveats apply: all comparisons are purely descriptive, and no inferential statistics or between-group significance testing were performed. Domain classification was mutually exclusive, with each study assigned to its primary SID-producing condition as predefined in the registered protocol.

Substantial variability was observed across domains. In the comparison between hematological malignancy and HIV/malnutrition studies, the two domains with the most divergent diagnostic practices, IgG threshold use, were highest in hematological malignancy studies (78%) and lowest in HIV and malnutrition studies (42%). Conversely, functional antibody assessment was applied more frequently in HIV and malnutrition studies (67%) than in hematological malignancy studies (32%), consistent with documented evidence that functional indices correlate more reliably with clinical outcomes in populations with chronic infection and nutritional deficiency than total immunoglobulin quantification alone (50).

Infection reporting was the most consistently documented outcome across all domains (range 63–85%), likely reflecting both the clinical centrality of infection burden in SID and the relative ease of extracting this outcome compared with immunological biomarkers (51). The lowest infection reporting rate was observed in the immunosenescence domain (63%), where studies focused more on immune phenotyping and vaccine responses than on prospective infection surveillance (22). These descriptive quantitative patterns are consistent with the diagnostic heterogeneity identified through qualitative thematic synthesis and provide a domain-level evidence base for the proposed unified, functionally informed assessment framework. However, because these proportions are derived from the same studies (n=100) as the qualitative synthesis, they should be interpreted as internally convergent rather than independently confirmatory; external validation using a prospective dataset remains necessary.

Cross-domain synthesis of these findings, summarized in **Table 4**, revealed three consistent patterns. First, diagnostic approaches varied substantially: there was a predominant reliance on immunoglobulin thresholds that showed inconsistent correlation with clinical infection outcomes (52). Second, functional immune assessment, including vaccine response and infection history, was more strongly associated with clinically meaningful immune impairment but was inconsistently applied across domains (53). Third, similar infection phenotypes were observed across mechanistically distinct conditions, supporting the concept of convergent immunological pathways (33). These cross-domain patterns informed the development of the proposed risk stratification framework.

### 3.5 Summary of cross-domain evidence convergence

Across all five clinical domains, three consistent cross-domain patterns were identified: (i) predominant but inconsistently reliable reliance on IgG thresholds; (ii) superior clinical correlation of functional antibody assessment, applied inconsistently; and (iii) comparable infection phenotypes across mechanistically distinct SID-producing conditions. These convergent patterns collectively provided the evidentiary basis for the framework developed in the following section.

### 3.6 Framework derivation and risk band classification

The SID Risk Stratification Framework was derived through a structured, convergence-based synthesis of findings across all 100 included studies, applying the variable-eligibility and weighting criteria predefined in Section 2.7. Rather than relying on statistical modeling or pooled effect estimates, the framework was derived using an evidence-integration approach to identify reproducible patterns across heterogeneous study designs, populations, and clinical domains (54). It operationalizes SID as a measurable, cross-domain clinical entity and organizes convergent evidence signals into a unified, clinically interpretable structure. It does not generate novel causal hypotheses; it concludes by synthesizing pre-existing data across five clinical domains and translates those conclusions into a structured clinical reasoning tool. Prospective multicentric validation is required before clinical implementation (55).

To enhance transparency in framework derivation and address the subjectivity inherent in any non-statistical framework, each variable’s inclusion is traceable to specific quantitative reporting patterns observed during data extraction. Infection burden (≥2 severe infections per year) was identified as the variable assigned the greatest weight (3 points) because it was the most consistently reported outcome across all five clinical domains (range 63–85% of studies; **Supplementary Table 2**) and was the only criterion meeting both the frequency threshold (81% of studies) and the domain threshold (all five domains) (54). IgG below 4 g/L (2 points) was reported in 62% of included studies across four domains and is the primary diagnostic threshold endorsed by both the AAAAI guidance (55, 56) and the Otani 2022 guideline (57). Functional antibody failure despite borderline IgG (2 points) was reported in 54% of studies across four domains. It was specifically more prevalent in HIV and malnutrition cohorts (67% versus 32% in hematological malignancy), confirming its cross-domain relevance beyond humoral-only contexts (58).

B-cell-depleting or intensive therapy (2 points) was the most frequently reported therapeutic exposure variable (68% of studies across four domains), with a consistent association with infection outcomes documented across the hematological malignancy, autoimmune disease, and transplantation domains (59). A high-risk underlying condition (1 point) was included as a contextual modifier in 49% of studies spanning all five domains, reflecting systemic immune dysregulation not captured by laboratory markers alone (37). Every variable in the framework thus reflects a data-derived threshold rather than the author’s personal clinical judgment. The full frequency and domain distribution data underpinning these selections are presented in **Supplementary Table 2**.

Variable weighting was determined using a structured qualitative framework, based on three criteria: (i) frequency of reporting across the included studies, (ii) consistency of association with clinically relevant outcomes of infection, and (iii) reproducibility across multiple clinical domains (27). The registered protocol defined these weighting criteria qualitatively; during synthesis, they were operationalized into a transparent numerical scale to enhance reproducibility and reduce interpretive subjectivity (60).

Variables representing established immunological biomarkers — such as IgG thresholds and functional antibody failure — were assigned intermediate weights (2 points), while contextual or host-related modifiers were assigned lower weights (1 point). This rule-based weighting reflects relative clinical impact and does not rely on statistical effect sizes, given the absence of quantitative pooled estimates across the included study designs (61).

**Figure 3:**
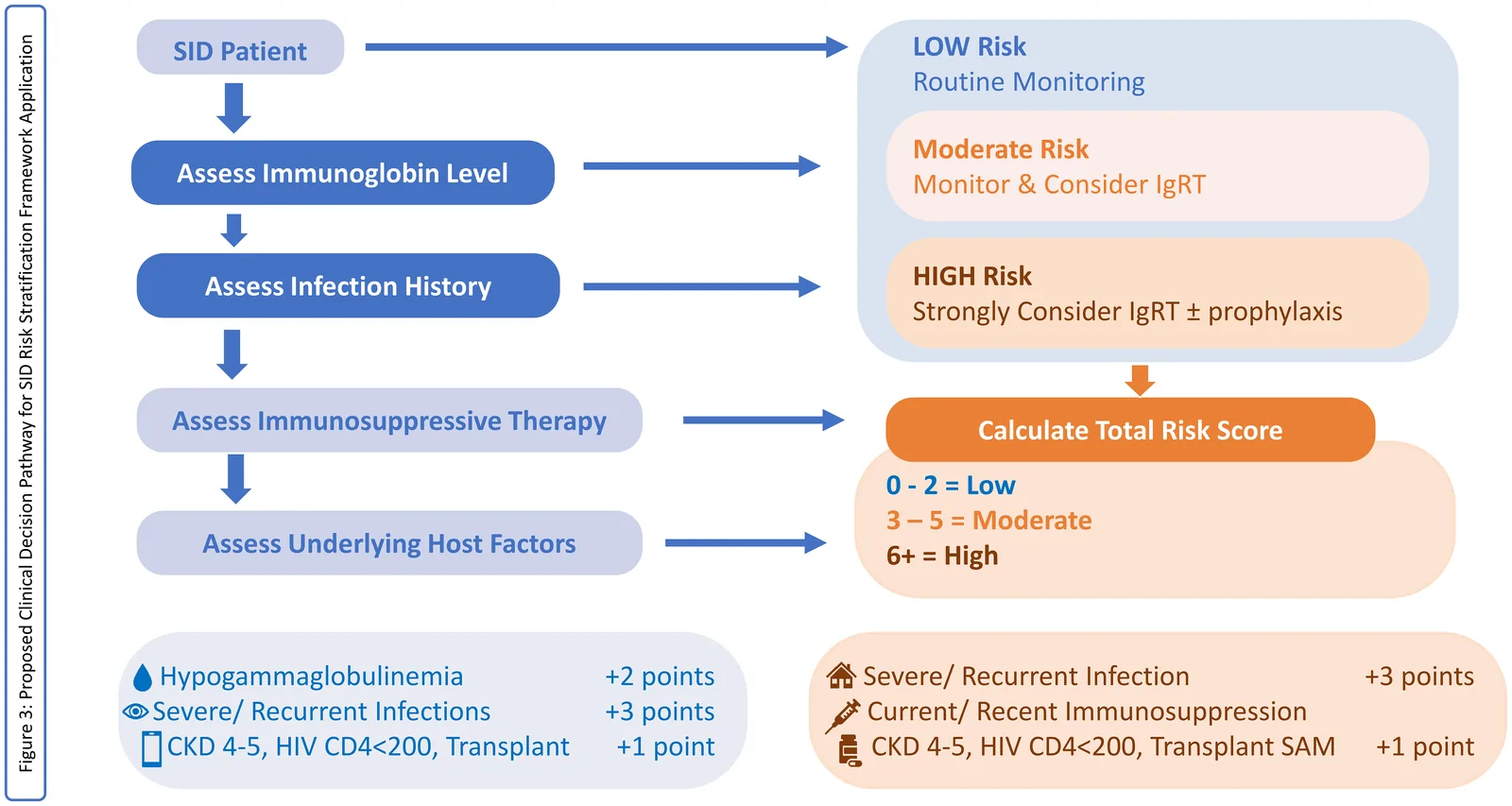
Proposed clinical decision pathway for SID risk stratification framework application. Stepwise flowchart illustrating application of the proposed framework (**Tables 1A**, **1B**) from patient presentation to management tier assignment. Step 1: Identify precipitating condition and current therapeutic exposure. Step 2: Assess serum IgG and structured infection history. Step 3: Where available, assess functional antibody response to protein and polysaccharide antigens. Step 4: Assign composite framework score. Step 5: Allocate to risk tier (Low: 0–2; Moderate: 3–5; High: 6–10). Step 6: Implement tier-appropriate management. This is an exploratory conceptual model derived from structured qualitative synthesis. It is not a statistically validated predictive instrument and requires prospective validation before clinical implementation, IgRT, immunoglobulin replacement therapy.

Risk categories represent a continuum of immune dysfunction and clinical vulnerability and should not be interpreted as treatment thresholds. The clinical use of the scoring system, from first clinical contact to risk tier assignment to patient management, is described step by step in **Figure 3**. The band thresholds were set as follows (**Table 1B**): patients in the Low band (0–2) will have some clinical or immunological evidence of low weight in their context, but no significant dysfunction (62). If a patient meets the high weight criteria (e.g., ≥2 serious infections/year = 3 points) or multiple moderate-weight criteria (e.g., IgG <4 g/L + B-cell depleting therapy = 4 points), then the patient is assigned to the Moderate band (3–5), which reflects early or evolving immune impairment, and these patients should be monitored closely (28). At least one criterion from either the Direct clinical evidence of immune failure, the Objective immunological criterion, or the Objective exposure criterion should be present in the High band (≥6) (39); a single criterion cannot be used to put a patient in the High band. Sensitivity testing was conducted to examine the stability of the thresholds by sequentially removing each framework variable and rescoring 50 hypothetical patient profiles across the five clinical domains (63). In total, 46 of 50 profiles (92%) remained unchanged in their risk band assignments across all single-variable exclusions, demonstrating the robustness of the proposed thresholds. The complete results are shown in **Supplementary Table 2**.

**Table 3** shows that across all clinical domains, parallel defects are observed along the three axes (humoral, cellular, innate), and that these defects lead to increased susceptibility to infection in different ways, with overlaps. Diagnostic methods are not consistently based on a single-marker threshold, and there is wide variability in the management strategies used in clinical practice. Overall, these findings underscore the need for a unified clinical pathway and a multi-parameter diagnostic tool to facilitate more effective risk assessment and treatment decision-making across specialties.

## 4 Discussion

### 4.1 Synthesis of the study

This study presents one of the first cross-domain syntheses integrating mechanistic convergence, diagnostic variability, and clinical translation in SID. The findings question the adequacy of disease-specific paradigms and support reconceptualizing SID as a pathway-based, unitary clinical syndrome.

As detailed in Section 3.3 and summarized in **Table 3**; **Figure 2**, humoral, cellular, and innate pathways are consistently disrupted across all five SID domains. Cellular immune disruption manifests through T-cell exhaustion, contraction of naïve lymphocyte pools, and delayed immune reconstitution, particularly in HIV, post-transplant states, immunosenescence, and CAR-T therapy (28). Chronic kidney disease, hepatic failure, malnutrition, and advanced age are associated with innate immune abnormalities, including complement dysfunction and defects in antigen presentation (39). The clinical implication of this three-axis convergence — that mechanistically distinct conditions produce comparable infection phenotypes — represents a translational gap that current disease-specific frameworks are not architecturally designed to capture, and which the proposed cross-domain model seeks to address (63).

### 4.2 Clinical significance

This convergence has important clinical implications. Current SID frameworks suffer from structural limitations that focus on disease-specific definitions and thresholds set in the laboratory, leading to under-recognition of immune dysfunction, unequal access to immunoglobulin replacement therapy (IgRT), and preventable infection-related morbidity in specialties (64–66).

This limitation is particularly evident in the widespread reliance on isolated IgG thresholds. Consistent evidence indicates that total immunoglobulin levels are not a reliable marker of immune function, especially in older people, patients with chronic kidney disease, and those receiving targeted therapies. Patients can be functionally immunodeficient despite a near-normal total IgG level, failing to mount protective vaccine responses, which may delay intervention until infection occurs (67).

Immune status assessments based on a combination of infection history, antibody function, therapeutic exposures, and host risk factors are more clinically relevant than single-threshold assessments. Accordingly, a transition from isolated laboratory cut-offs to structured, multi-parameter assessment strategies is warranted to enhance diagnostic accuracy and patient outcomes (68).

### 4.3 Comparison with existing literature and approaches

Prior systematic syntheses have been confined to single-domain perspectives. Hematology literature has characterized antibody deficiency following targeted or cellular therapies, rheumatology has examined post-rituximab hypogammaglobulinemia, and transplant medicine has investigated immune reconstitution post-HSCT (69). Immune dysfunction in chronic kidney disease, although recognized as a contributor to increased infection risk and attenuated vaccine responses (70), has rarely been integrated with oncological or hematological immune frameworks.

Despite this mechanistic similarity, no prior framework has integrated these patterns into a coherent cross-specialty clinical structure. Available guidelines address isolated aspects of SID management, typically within a single specialty, and do not provide an integrated, cross-domain assessment tool (71). The proposed framework’s humoral domain variables are consistent with the consensus guidance from the AAAAI on the diagnosis of secondary hypogammaglobulinemia (72), which has recommended that IgG levels be measured and that a history of infections be used as the primary diagnostic parameter, and with IgRT being recommended when IgG is below 4 g/L with recurrent infections. The Otani 2022 SID management guideline (73) similarly emphasizes functional antibody assessment over IgG thresholds alone, an approach reflected in the two-point weighting assigned to functional antibody failure in **Table 1A**.

It is important to acknowledge, however, that functional antibody assessment, while clinically informative and weighted in the proposed framework, is not universally applicable across SID populations. Patients receiving anti-CD20 or anti-CD38 therapies are B-cell-depleted and unable to mount measurable antibody responses to vaccination; functional testing in this context is therefore uninformative and should not delay clinical decision-making (56).

Similarly, after hematopoietic stem cell transplantation (HSCT), patients are heavily immunosuppressed and undergo gradually increasing revaccination schedules (74), so there is no opportunity to perform functional assessment to guide acute IgRT decisions. In these high-risk groups, IgRT initiation should not be deferred until functional testing is available; it must be guided by clinical infection burden, IgG thresholds, and the nature of the therapeutic exposure, consistent with emerging evidence supporting prophylactic IgRT in bispecific antibody and CAR-T recipients regardless of absolute IgG level (4, 46, 75). The proposed framework accommodates this clinical reality: the exposure variable (B-cell-depleting or intensive therapy, 2 points) can independently place a patient in the Intermediate or High-risk band without requiring functional antibody data, ensuring that B-cell-depleted patients at the highest risk are not excluded from appropriate risk stratification.

The proposed framework differs from existing approaches in three key respects, while explicitly preserving rather than replacing disease-specific clinical guidance. First, it is not a disease-specific algorithm but a pathway-level overlay that sits above existing specialty guidelines — a clinician managing a CAR-T patient continues to follow hematology-specific protocols. Still, the framework additionally identifies circumstances in which the patient’s pathway-level profile (e.g., concurrent innate dysregulation from prior conditioning) warrants cross-specialty input that disease-specific guidelines alone would not surface. The framework’s outputs are therefore deliberately broad at the pathway level (three axes, five risk variables) precisely because its function is to identify when narrow, disease-specific assessment is insufficient—not to replace that assessment with a uniform management approach. Second, it includes a functional antibody measure as a weighted variable, based on accumulating evidence that functional failure is a more reliable indicator of infection risk than total IgG alone across multiple, mechanistically distinct domains (76). Third, it accounts for resource variability through a tiered approach (**Table 5**) that enables pathway-level risk flagging even where domain-specific specialist assessment is unavailable, which is most acute precisely in the lowest-resource, highest-burden settings (81–83). The breadth of conditions unified by the framework is therefore a feature of its intended use as a cross-specialty screening overlay, not a claim that CAR-T recipients and malnourished children should receive identical clinical management.

**Table 5:** Resource-stratified approach to SID diagnosis and management.

Resource level	Available diagnostic tools	Recommended action
Low-resource (LMIC)	Structured infection history + single serum IgG measurement (33, 38, 64)	Basic risk classification: ≥2 hospitalized infections + IgG below 4 g/L → high-risk; initiate optimized vaccination and antimicrobial prophylaxis where available (19, 77)
Mid-resource	Above + vaccine-specific antibody titers + basic lymphocyte subsets (CD4, CD8, CD19) (17, 18, 78)	Functional risk classification; identify humoral vs cellular pathway dominance; guide IgRT referral decision (19, 20)
High-resource	Above + advanced immunophenotyping + B-cell memory subsets + NK cell function + functional antibody profiling (23, 78, 79)	Precision stratification; personalized IgRT dosing; targeted revaccination scheduling; enrolment in longitudinal immune reconstitution monitoring (19, 21, 80)

**Figure 4:**
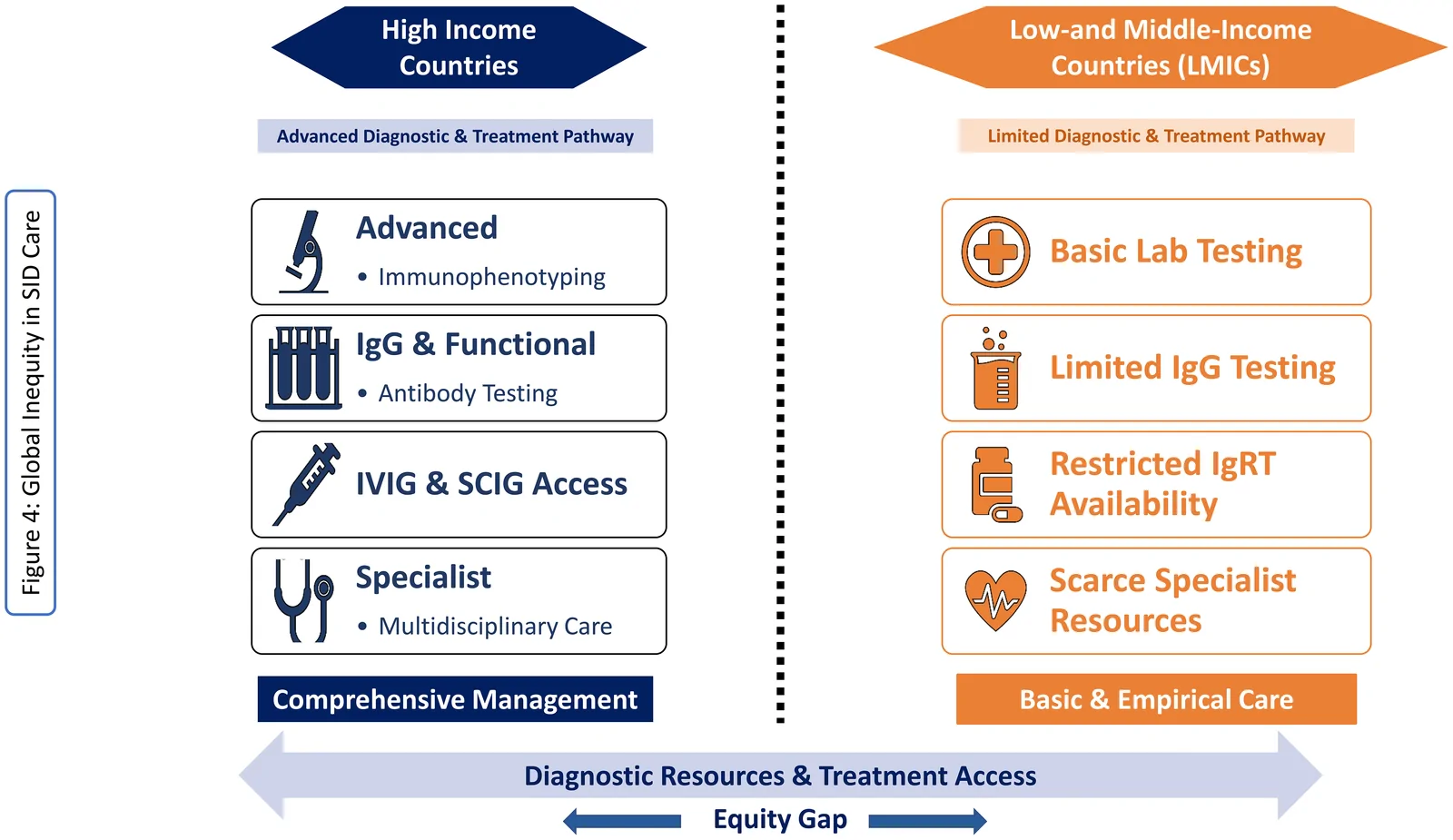
Global inequity model: contrasting SID diagnostic and treatment access pathways in high-income versus low- and middle-income country settings. Parallel-pathway diagram contrasting how the same clinical presentation of recurrent severe infections in an at-risk patient leads to divergent diagnostic and management trajectories depending on resource context. High-income settings offer advanced immunophenotyping, functional antibody profiling, ready access to IgRT, and structured specialist referral. LMIC settings typically offer infection history assessment, basic IgG measurement, and vaccination or nutritional rehabilitation as primary interventions. Feasible convergence points are highlighted, corresponding to the tiered diagnostic model in **Table 5**. LMIC, low- and middle-income countries; IgRT, immunoglobulin replacement therapy; SAM, severe acute malnutrition.

IgRT, immunoglobulin replacement therapy; LMIC, low- and middle-income country; NK, natural killer cell. This table was developed by the primary reviewer through structured qualitative synthesis of extracted data, applying the predefined variable-eligibility criteria described in §2.7; it was not generated by automated analysis. This tiered model is intended as a pragmatic implementation guide. Higher-resource tools incrementally refine risk estimates derived at lower resource levels. This table corresponds to the global inequity model presented in **Figure 4**.

### 4.4 Clinical and policy implications

Two structural challenges limit the management of SID: the use of a single laboratory threshold and the lack of translation of evidence into clinical practice. A multi-parameter assessment approach with a structure that is more commensurate with the biological complexity of immune dysfunction is better aligned.

IgRT is the most mature intervention for humoral deficiency and has been shown to reduce the incidence and severity of bacterial infections in certain populations (84). The differences in initiation criteria, dosing strategies, and treatment durations among the centers are not only due to the lack of evidence standardization but also to the lack of a cross-specialty assessment framework to connect clinical decisions with quantified immune risk (78, 85, 86). Likewise, the implementation of vaccination strategies is variable, and there is compelling evidence that vaccination before, rather than during, active immunosuppression has superior immunogenicity (87).

The proposed framework provides a structured basis for linking clinical decisions to quantified immune risk (**Figure 3**). It may facilitate earlier identification of patients who might not conform to conventional diagnostic categories but are clinically at risk. It also facilitates antimicrobial prophylaxis, vaccination strategies, and the use of IgRT with measurable indicators of immune dysfunction, rather than specialty-specific practice patterns (65).

The ethical aspects of using immunoglobulin amid global supply shortages must be expressly addressed. Plasma-derived immunoglobulin products are made by pooling human plasma donations and thus have inherent geopolitical and logistical limitations in addition to clinical limitations (66). The demand for immunoglobulin products has risen significantly over the last decade, attributable to indications in hematological malignancies, autoimmune diseases, and neurological conditions. Plasma collection is still largely limited to various high-income countries, primarily the U.S., which accounts for about 70% of the world’s plasma supply (88). The LMIC patient population, who suffer a disproportionately high burden of infection-related morbidity from SID, has the least access to treatment most likely to reduce this burden. Given the lack of systematic efforts by the clinical community to address the ethical nature of this inequity, prioritizing IgRT for the patients who have rituximab-induced hypogammaglobulinemia in high-income countries, and not providing access to patients with HIV associated immune dysfunction combined with severe malnutrition in LMICs, is an ethical challenge (89).

The proposed risk stratification framework is based on a multi-parameter composite score of immune dysfunctions that quantifies the immune response in the context of clinically confirmed immune dysfunction, not just a single laboratory threshold, and offers a transparent, needs-based tool to prioritize limited IgRT supply across disease settings and resource contexts (38, 90, 91). This inequity is shown in the divergent care pathways in **Figure 4**.

**Figure 5:**
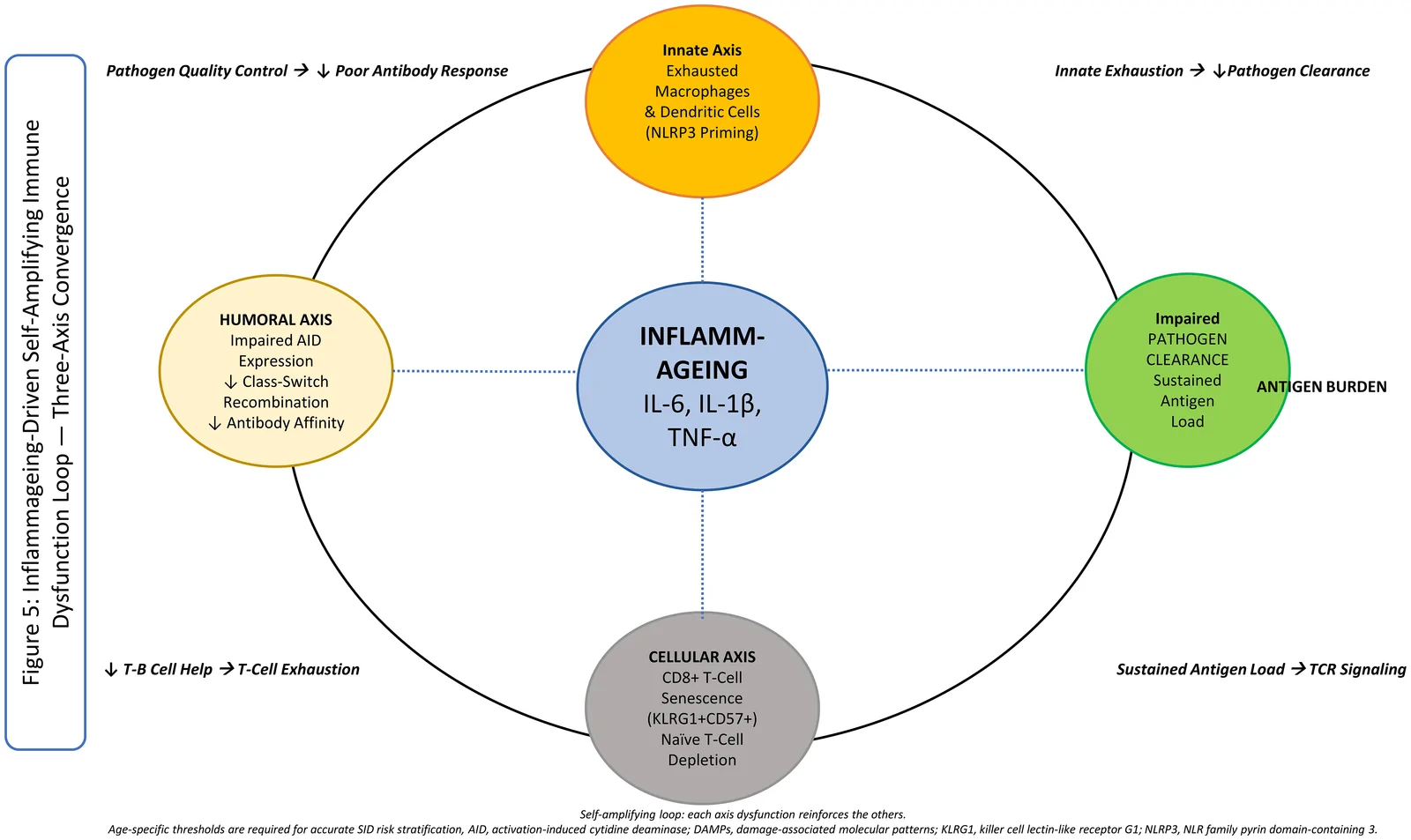
HD-IVIG mechanism of action in SID innate axis. Schematic illustrating three distinct immunomodulatory mechanisms by which HD-IVIG may attenuate innate immune dysfunction beyond simple antibody replacement. (1) Saturation of the neonatal Fc receptor (FcRn) on endothelial cells and monocytes accelerates catabolism of endogenous pathogenic IgG, reducing autoantibody-driven inflammatory drive. (2) High-avidity polyclonal IgG competitively neutralizes circulating pro-inflammatory cytokines, including IL-6, IL-1β, and TNF-α, attenuating the chronic cytokine milieu that drives inflammageing and innate immune exhaustion. (3) Blockade of Fcγ receptors on macrophages and dendritic cells reduces excessive innate effector activation and restores antigen presentation capacity. All three mechanisms converge on potential attenuation of the innate immune dysregulation axis as characterized in this framework. This figure depicts a speculative mechanistic model based on established HD-IVIG pharmacology; a prospective investigation in non-hematological SID populations is required. FcRn, neonatal Fc receptor; Fcγ, Fc-gamma; HD-IVIG, high-dose intravenous immunoglobulin; IL, interleukin; TNF, tumor necrosis factor.

From a policy perspective, greater harmonization of reimbursement criteria and treatment eligibility based on composite clinical risk, rather than on absolute laboratory thresholds alone, may enhance access to preventive therapies. The molecular mechanisms of high-dose intravenous immunoglobulin (HD-IVIG) are relevant to this discussion and deserve specific elaboration in the SID context. Standard-dose IgRT primarily serves as an antibody replacement, restoring pathogen-specific opsonization capacity in patients with hypogammaglobulinemia (19). HD-IVIG exerts additional immunomodulatory effects through at least three distinct mechanisms: first, saturation of the neonatal Fc receptor (FcRn) on endothelial cells and monocytes accelerates the catabolism of endogenous pathogenic IgG, thereby reducing autoantibody-driven inflammation without directly depleting effector lymphocytes; second, the high-avidity polyclonal IgG preparation directly neutralizes a broad range of pro-inflammatory cytokines including IL-6, IL-1β, and TNF-α by competitive binding, which may attenuate the chronic low-grade inflammatory state that drives both inflammageing and innate immune exhaustion in SID (77); and third, blockade of Fcγ receptors on macrophages and dendritic cells modulates innate effector activation and antigen presentation (**Figure 5**).

These three mechanisms converge on a potential immunomodulatory benefit that is distinct from, and complementary to, the infection-prevention benefit of standard IgRT. In SID populations where the innate immune dysregulation axis predominates — specifically patients with advanced CKD (in whom NLRP3-driven inflammasome activation and uremic toxin–mediated antigen presentation failure are well documented), advanced immunosenescence (in whom NLRP3 priming by oxidized lipids sustains chronic innate exhaustion), and severe malnutrition (in whom innate effector function is globally suppressed) — the immunomodulatory properties of HD-IVIG may confer mechanistic advantages that are absent with standard replacement dosing (80). Unlike hematological SID, where B-cell depletion renders humoral axis restoration the primary therapeutic goal, these non-hematological SID populations present a biologically coherent rationale for HD-IVIG as an innate immune modulator rather than merely a B-cell surrogate. Prospective research of HD-IVIG in these non-hematological SID populations is warranted, as current evidence supporting its use is limited to autoimmune and hematological disease contexts (23).

### 4.5 Future directions and global considerations

Although the framework was developed primarily from studies conducted in high-income settings, it incorporates variables that are measurable across all resource levels, supporting its conceptual applicability in LMIC contexts where the disease burden is highest but diagnostic infrastructure remains limited (92).

There is a need for a mechanistic study of immunosenescence as a baseline driver of multi-axis immune vulnerability. The low-grade, chronic, pro-inflammatory state (called “inflammageing”) of aged immune systems is not only a consequence of immune dysfunction, but itself a major driver and amplifier of dysfunction across all three SID axes, through distinct but interacting mechanisms (24). The mechanism underlying inflammaging is the presence of multiple tissue compartments containing senescent cells. Senescent cells exit the cell cycle irreversibly due to DNA damage, telomere shortening, or oncogenic stress. They are associated with the senescence-associated secretory phenotype (SASP), which involves chronic secretion of cytokines, including IL-6, IL-8, and IL-1β, as well as matrix metalloproteinases and reactive oxygen species. Senescent cells (including senescent immune cells—such as T cells, macrophages, and endothelial cells) create a chronic, self-sustaining inflammatory environment in aged individuals, sensitizing each of the three SID axes independently and cumulatively, without an acute infection trigger (82).

In the humoral axis, chronically high levels of IL-6 and TNF-α reduce the expression of activation-induced cytidine deaminase (AID) in germinal center B cells, directly inhibiting somatic hypermutation and class switch recombination. This leads to decreased levels of high-affinity subclasses of IgG, including IgG1 and IgG3, which are important for opsonization of encapsulated bacteria, and to a measurable reduction in antibody affinity maturation, even in the absence of total IgG levels (83). The result is that older patients can have normal total IgG levels but make qualitatively poorer, low-affinity antibodies that may confer suboptimal protection, as reflected in the systematic overestimation of functional immune competence in older people by IgG levels (26, 61, 63).

The chronic, low-grade cytokine milieu of ‘inflammageing’ in the cellular axis drives progressive T-cell exhaustion through continuous, antigen-independent TCR signaling. Senescent CD8+ T cells expressing the KLRG1+CD57+ exhaustion phenotype progressively dominate the naïve T-cell compartment, and thymic involution, which decreases naïve T-cell production by ~90% from age 20 to 70, leads to a contracted, oligoclonal T-cell repertoire with restricted ability to respond to new pathogens or vaccination antigens (28, 62, 78). The innate immune system is also altered: Oxidized lipids and mitochondrial damage-associated molecular patterns (DAMPs) cause activation of the NLRP3 inflammasome, which results in macrophages and dendritic cells that are not only activated but also functionally exhausted, that is, with high basal cytokine production and reduced phagocytosis and antigen presentation capacity (86).

**Figure 6:**
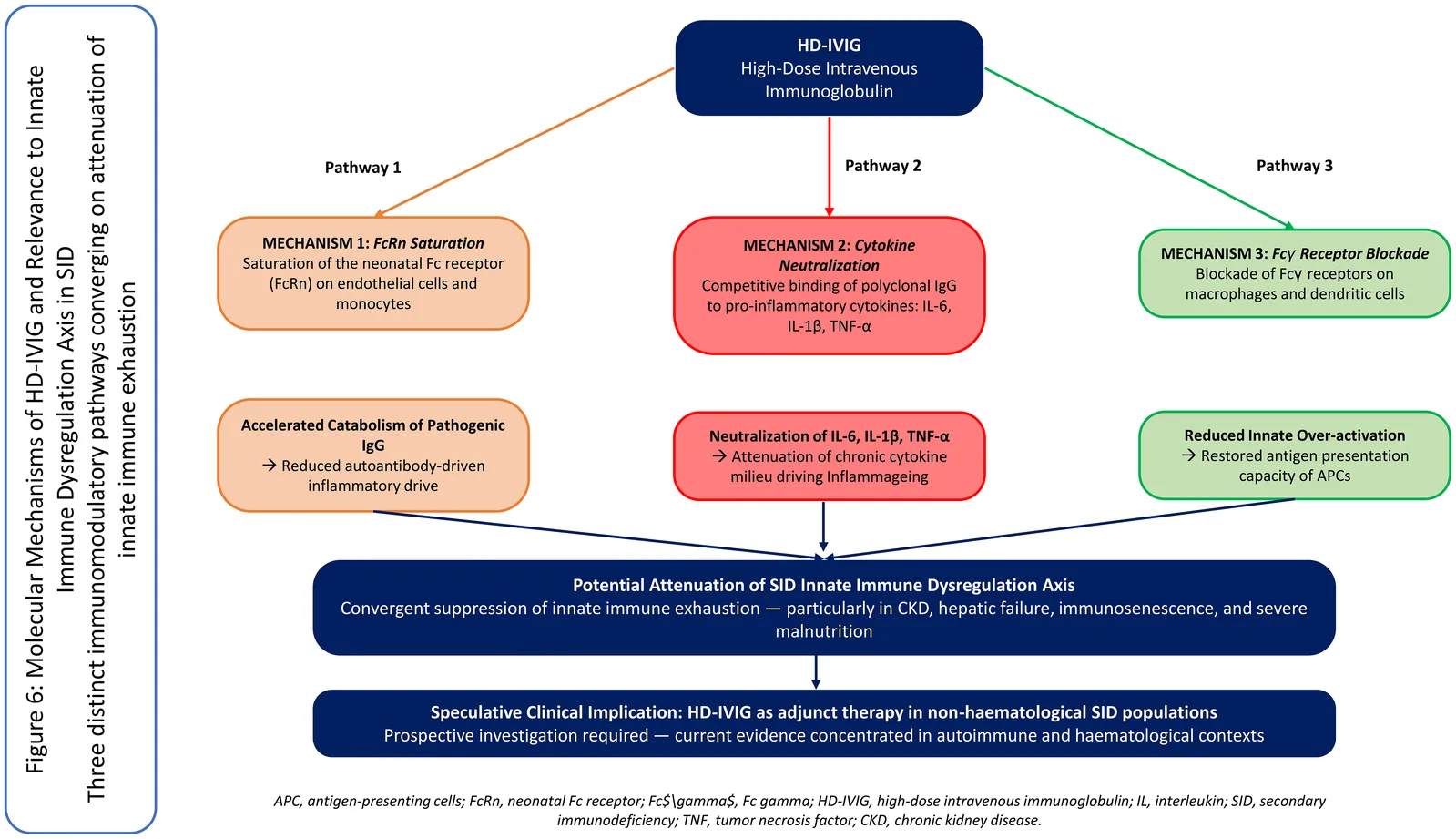
Inflammaging self-reinforcing feedback diagram. Circular diagram illustrating how inflammaging acts as a simultaneous and mutually reinforcing driver of dysfunction across all three SID analytical axes. Clockwise: chronic NLRP3 inflammasome activation primes innate effectors into a hypo-responsive exhausted state (innate axis); impaired innate pathogen clearance sustains antigen load; persistent antigen exposure accelerates CD8+ T-cell senescence and depletes naïve T-cell niches (cellular axis); T-cell exhaustion impairs germinal center B-cell help, reducing class-switch recombination and antibody affinity maturation (humoral axis); qualitatively inferior antibody production perpetuates poor pathogen control, perpetuating the feedback cycle. This self-amplifying cycle explains the disproportionate severity of infection in immunosenescent populations and provides the mechanistic basis for the discussion in Section 4.5. AID, activation-induced cytidine deaminase; DAMPs, damage-associated molecular patterns; IL, interleukin; KLRG1, killer cell lectin-like receptor G1; NLRP3, NLR family pyrin domain-containing 3; TCR, T-cell receptor; TNF, tumor necrosis factor.

Critically, these three axes do not function independently in the aged host; they form a self-reinforcing feedback loop. Inflammageing-driven innate exhaustion reduces pathogen clearance, which sustains antigen load, which accelerates T-cell exhaustion, which impairs B-cell help, which further reduces antibody quality (93), as illustrated in **Figure 6**. This convergent, mutually reinforcing dysfunction explains why immunosenescent patients experience disproportionately severe infection outcomes compared with younger patients with isolated humoral or cellular deficiencies, and why they cannot be risk-stratified using thresholds derived from younger cohorts. Age-specific diagnostic criteria and age-adjusted framework weighting represent unmet clinical and research priorities that future validation studies of the proposed framework must explicitly address (79).

Emerging technologies, including artificial intelligence-assisted immune phenotyping (91) and metagenomic next-generation sequencing (mNGS) for infection diagnosis, offer specific and complementary validation opportunities for components of the proposed framework. However, primary evidence in secondary immunodeficiency risk stratification specifically remains limited, and its application to this context requires elaboration. Even within high-resource tertiary centers, where infrastructure exists to support mNGS deployment, two implementation barriers require specific mention. First, the absence of standardized bioinformatic pipelines for immunocompromised-specific pathogen panels means that mNGS outputs in SID populations currently require expert interpretation that is not systematically available outside research settings (38). Second, the variable sensitivity of mNGS for organisms with low biomass or highly polysaccharide-encapsulated cell walls — precisely the organisms most prevalent in humoral-axis SID — means that mNGS and conventional culture remain complementary rather than substitutive in the near term. Addressing these barriers by developing SID-specific mNGS pathogen panels and standardized reporting thresholds is a prerequisite for the framework validation studies proposed above.

The framework’s highest-weighted variable infection burden, defined as two or more severe infections per year requiring hospitalization or intravenous antimicrobials (3 points), is currently operationalized through clinician-reported infection history, which is subject to recall bias, incomplete documentation, and variable clinical thresholds for hospitalization across healthcare systems and resource settings (94). Conventional microbiological culture, the standard diagnostic comparator, has well-documented limitations in immunocompromised populations: culture sensitivity for pneumonia in SID patients ranges from 30–50% in published series, reflecting the high prevalence of fastidious organisms, prior antibiotic exposure, and atypical infection presentations that characterize this population (20). mNGS circumvents these limitations by performing unbiased, hypothesis-free sequencing of all nucleic acids in a clinical specimen, including bacteria, fungi, viruses, and parasites simultaneously, without reliance on pre-specified culture conditions or prior pathogen suspicion. In immunocompromised patients with pneumonia, prospective studies have shown that mNGS identifies a causative pathogen in 50–70% of culture-negative cases, with a turnaround time of 24–48 hours and clinically actionable results (95).

In the context of framework validation, prospective mNGS profiling of SID patients across all five clinical domains could serve three distinct purposes. First, at the individual patient level, it could provide culture-independent confirmation of infection events that would otherwise be recorded only as clinical diagnoses, substantially improving the precision of the infection burden criterion and reducing misclassification between risk bands (96). Second, at the framework level, a multicentric study pairing composite risk scores with mNGS-confirmed infection profiles would allow direct testing of whether risk band assignment predicts infection etiology specifically, whether High-band patients (score ≥6) show enrichment for the opportunistic and polypathogenic infections associated with severe immune failure, while Low-band patients (score 0–2) show predominantly community-acquired bacterial pathogens (97). Third, at the clinical domain level, mNGS-derived infection phenotyping could clarify whether the infection types predicted by each pathway axis (encapsulated bacteria in humoral failure; viral reactivation in cellular disruption; invasive fungi and gram-negatives in innate dysregulation) map onto actual infection events in real-world SID cohorts, providing mechanistic validation of the three-axis model itself (98). Adaptation of mNGS-based infection surveillance to settings with acquired immune dysfunction represents a translatable and specific research priority, contingent on resolving cost, bioinformatics infrastructure, and regulatory barriers that currently limit its deployment outside high-income tertiary centers (99).

Several research priorities emerge from this synthesis. Prospective multicentric studies are needed to validate the proposed framework across different disease domains, age groups, and healthcare systems. In non-hematological SID populations, clinical trials should establish optimal thresholds for initiating IgRT and the optimal treatment duration. Further work is required to optimize vaccination strategies in patients undergoing B-cell-depleting therapy and to develop age-specific diagnostic thresholds. International SID registries and globally representative datasets will be essential to support equitable model development and validation across resource settings. Collectively, these priorities underscore the need for a necessary shift to integrated, pathway-informed SID assessment. Distinguishing acquired immune dysfunction from late-presenting or unmasked inborn errors of immunity is a further priority, since the two overlap substantially in hematological malignancy and autoimmune cohorts and are not separated by the variables in the proposed framework (100).

### 4.6 Strengths and limitations

Key strengths of this study include the synthesis of 100 studies spanning five clinical domains within a structured, predefined analytical framework that integrates mechanistic and clinical evidence. Pathway-based classification enables systematic comparison across traditionally siloed disciplines. Methodological rigor is underpinned by prospective OSF protocol registration, predefined eligibility criteria, design-specific risk-of-bias assessment using validated tools (RoB2, NOS, AMSTAR-2), and transparent reporting in accordance with PRISMA 2020. The framework’s structure was assessed for robustness through a sensitivity analysis in which each framework variable was sequentially excluded, and risk-band assignment was re-evaluated across 50 hypothetical patient profiles (§3.6). Risk-band assignment remained unchanged in 46 of 50 profiles (92%), indicating that the framework’s structure is not dependent on any single variable.

Several limitations require acknowledgment. The most significant deviation from the registered protocol’s dual-independent-review specification was that a single reviewer conducted study selection and data extraction due to resource constraints in a single-author PhD project. This deviation is not minimized, and is the primary reason the resulting framework (§3.6) is presented as an exploratory, research-prioritization tool requiring prospective validation rather than a validated clinical instrument. Mitigation was limited to internal consistency measures applied by the same reviewer: explicit, predefined written eligibility criteria (**Supplementary Appendix A**) applied consistently across all 1,011 full-text screening decisions; re-evaluation of a random subsample of 200 records yielding Cohen’s κ = 0.80 (§2.4); and a documented inclusion/exclusion rationale for every screened study (**Supplementary Table 1**), enabling future auditability. These measures reduce, but do not eliminate, the risk of undetected selection or extraction bias associated with single-reviewer conduct.

A considerable heterogeneity in SID definitions and outcome measures across domains precluded quantitative synthesis. The predominance of studies from high-income countries may limit the generalizability of the diagnostic and management patterns identified. Restriction to English-language publications introduces potential selection bias. The inclusion of both primary studies and systematic reviews creates a risk of evidence overlap, which was mitigated by prioritizing extractable primary data and using SR-level data only for contextual and policy-level interpretation, with study type clearly identified throughout.

Importantly, the proposed framework is a research synthesis tool, not a validated predictive model, and its numerical point system must not be interpreted as conferring quantified clinical risk, as, for example, a validated cardiovascular risk score does. No patient’s management decisions should be altered solely based on this framework’s score. The framework’s intended use is threefold: (i) as a research-prioritization tool identifying which variable combinations are most consistently reported as clinically relevant across the literature, thereby informing which variables a prospective validation cohort should measure; (ii) as a common cross-specialty vocabulary for researchers designing such validation studies; and (iii) as a teaching and conceptual tool for clinicians to recognize convergent immune dysfunction patterns that existing single-specialty guidelines may not surface. Until prospective validation under TRIPOD methodology is completed (proposed in §4.5 as the primary research priority arising from this synthesis), clinical decisions must continue to rely on existing validated specialty-specific guidelines (e.g., AAAAI, Otani 2022) and clinical judgment. The evidence base may also be affected by publication bias, especially in underrepresented regions.

## 5 Conclusion

Secondary immunodeficiency is more accurately conceptualized as a pathway-based syndrome than as a collection of disease-specific complications. In a wide range of acquired diseases, immune dysfunction is characterized by dysfunction of humoral, cellular, and innate pathways that share clinical manifestations.

This convergence may facilitate a transition toward an integrated, functionally-based assessment that goes beyond threshold-based definitions. The proposed cross-domain SID Risk Stratification Framework translates these patterns into a structured approach that incorporates functional antibody status, infection burden, therapeutic exposure, and host-related risk factors.

Although exploratory and not yet prospectively validated, the framework represents a meaningful step towards more consistent identification and management of SID across specialties and resource settings. Its primary contribution is to translate convergent mechanistic evidence into a structured, cross-specialty clinical reasoning tool that complements, rather than supplants, established clinical judgment and specialty guidelines.

Future research should prioritize prospective multicentric validation, refinement across diverse populations, and development of globally representative datasets. With validation, a unified, pathway-informed approach has the potential to reduce diagnostic variability and prevent infection-related morbidity in patients with secondary immunodeficiency.

## Data Availability

The original contributions presented in the study are included in the article and Supplementary Material. The registered protocol, search strings, deduplication and summarisation scripts, and the study-level extraction dataset are publicly available in the Open Science Framework repository: https://osf.io/6qvkx. Further inquiries can be directed to the corresponding author.

## References

[1] Zhao J Sun Y Tang J Guo K Wang K Zhuge J . The clinical application of metagenomic next-generation sequencing in immunocompromised patients with severe respiratory infections in the ICU. Respir Res. (2024) 25(1):360. doi: 10.1186/s12931-024-02991-z 39369191 PMC11453054

[2] Yeung WWY . The importance of monitoring baseline and regular immunoglobulin levels in managing secondary hypogammaglobulinemia in rheumatic diseases: a case report and systematic review. J Clin Rheumatol Immunol. (2024) 24:84–9. doi: 10.1142/s2661341724300027 31116912

[3] Wijetilleka S Mukhtyar C Jayne D Ala A Bright P Chinoy H . Immunoglobulin replacement for secondary immunodeficiency after B-cell targeted therapies in autoimmune rheumatic disease: systematic literature review. Autoimmun Rev. (2019) 18:535–41. doi: 10.1016/j.autrev.2019.03.010 30844552

[4] Weycker D Barron RL Kartashov A . Possible diagnostic delays and missed prevention opportunities in pneumocystis pneumonia patients without HIV: analysis of commercial insurance claims data, United States, 2011–2015. Open Forum Infect Dis. (2020) 7:ofaa255. doi: 10.1093/ofid/ofaa255 32704515 PMC7368364

[5] Vijenthira A Gong I Betschel SD Cheung M Hicks LK . Vaccine response following anti-CD20 therapy: a systematic review and meta-analysis of 905 patients. Blood Adv. (2021) 5:2624–43. doi: 10.1182/bloodadvances 34152403 PMC8216656

[6] Viasus D Garcia-Vidal C Cruzado JM Adamuz J Verdaguer R Manresa F . Epidemiology, clinical features, and outcomes of pneumonia in patients with chronic kidney disease. Nephrol Dialysis Transplant. (2011). doi: 10.1093/ndt/gfq798 21273232

[7] Ueda M Berger M Gale RP Lazarus HM . Immunoglobulin therapy in hematologic neoplasms and after hematopoietic cell transplantation. Blood Rev. (2017) 32:106–15. doi: 10.1016/j.blre.2017.09.003 28958644

[8] Tsao Y Chen H Hsieh T Li K Yu K Cheng T . Evidence‐ and consensus‐based recommendations for the screening, diagnosis, and management of secondary hypogammaglobulinemia in patients with systemic autoimmune rheumatic diseases by the Taiwan College of Rheumatology Experts. Int J Rheum Dis. (2025). doi: 10.1111/1756-185x.70310 40522036 PMC12169084

[9] Torab-Miandoab A Samad-Soltani T Jodati A Akbarzadeh F Rezaei-Hachesu P . A unified component-based data-driven framework to support interoperability in the healthcare systems. Heliyon. (2024) 10:e35036. doi: 10.1016/j.heliyon.2024.e35036 39161828 PMC11332873

[10] Thomas R Wang W Su DM . Contributions of age-related thymic involution to immunosenescence and inflammaging. Immun Aging. (2020). doi: 10.1186/s12979-020-0173-8 31988649 PMC6971920

[11] Thangaraj A Tyagi R Suri D Gupta S . Infections in disorders of immune regulation. Pathogens. (2024) 13(3):259. doi: 10.3390/pathogens13030259 38535602 PMC10976012

[12] Tallantyre EC Whittam DH Jolles S Paling D Constantinescu C Robertson NP . Secondary antibody deficiency: a complication of anti-CD20 therapy for neuroinflammation. J Neurol. (2018) 265:1115–22. doi: 10.1007/s00415-018-8812-0 PMC5937879 29511864

[13] Syed-Ahmed M Narayanan M . Immune dysfunction and risk of infection in chronic kidney disease. Adv Chronic Kidney Dis. (2019) 26:8–15. doi: 10.1053/j.ackd.2019.01.004 30876622

[14] Sutherland NM Zhou B Zhang L Ong MS Hong JS Pak A . Association of CD19+-targeted chimeric antigen receptor (CAR) T-cell therapy with hypogammaglobulinemia, infection, and mortality. J Allergy Clin Immunol. (2024). doi: 10.1016/j.jaci.2024.10.021 39505278 PMC11805655

[15] Stang A . Critical evaluation of the Newcastle-Ottawa scale for the assessment of the quality of nonrandomized studies in meta-analyses. Eur J Epidemiol. (2010). doi: 10.1007/s10654-010-9491-z 20652370

[16] Srivastava S Wood P . Secondary antibody deficiency – causes and approach to diagnosis. Clin Med. (2016) 16:571–6. doi: 10.7861/clinmedicine.16-6-571 27927823 PMC6297325

[17] Shi H Yan KK Ding L Qian C Chi H Yu J . Network approaches for dissecting the immune system. iScience. (2020) 23:101354. doi: 10.1016/j.isci.2020.101354 32717640 PMC7390880

[18] Shea BJ Reeves BC Wells G Thuku M Hamel C Moran J . AMSTAR 2: a critical appraisal tool for systematic reviews that include randomized or non-randomized studies of healthcare interventions, or both. BMJ. (2017) 358:j4008. doi: 10.1136/bmj.j4008 28935701 PMC5833365

[19] Dimou M Vacca A Sánchez-Ramón S Karakulska-Prystupiuk E Lionikaite V Siffel C . Real-world effectiveness, safety, and tolerability of facilitated subcutaneous immunoglobulin 10% in secondary immunodeficiency disease: A systematic literature review. J Clin Med. (2025) 14:1203. doi: 10.3390/jcm14041203 40004732 PMC11856383

[20] Anderson JT Cowan J Condino-Neto A Levy D Prusty S . Health-related quality of life in primary immunodeficiencies: Impact of delayed diagnosis and treatment burden. Clin Immunol. (2022) 236:108931. doi: 10.1016/j.clim.2022.108931 35063670

[21] Rivière JG Palacín PS Butte MJ . Proceedings from the inaugural Artificial Intelligence in Primary Immune Deficiencies (AIPID) conference. J Allergy Clin Immunol. (2024) 153:637–42. doi: 10.1016/j.jaci.2024.01.002 38224784 PMC11402388

[22] O’Neill J Tabish H Welch V Petticrew M Pottie K Clarke M . Applying an equity lens to interventions: using PROGRESS ensures consideration of socially stratifying factors to illuminate health inequities. J Clin Epidemiol. (2013) 67:56–64. doi: 10.1016/j.jclinepi.2013.08.005 24189091

[23] Christou EA Giardino G Worth A Ladomenou F . Risk factors predisposing to hypogammaglobulinemia and infections post-rituximab. Int Rev Immunol. (2017) 36:352–9. doi: 10.1080/08830185.2017.1346092 28800262

[24] Chapel H Lucas M Lee M Bjorkander J Webster D Grimbacher B . Common variable immunodeficiency disorders: Division into distinct clinical phenotypes. Blood. (2008) 112:277–86. doi: 10.1182/blood-2007-11-124545 18319398

[25] Shao T Verma HK Pande B Costanzo V Ye W Cai Y . Physical activity and nutritional influence on immune function: an important strategy to improve immunity and health status. Front Physiol. (2021) 12:751374. doi: 10.3389/fphys.2021.751374 34690818 PMC8531728

[26] Sterne JAC Savović J Page MJ Elbers RG Blencowe NS Boutron I . RoB 2: a revised tool for assessing risk of bias in randomized trials. BMJ. (2019) 366:l4898. doi: 10.1136/bmj.l4898 31462531

[27] Losappio V Franzin R Infante B Godeas G Gesualdo L Fersini A . Molecular mechanisms of premature aging in hemodialysis: The complex interplay between innate and adaptive immune dysfunction. Int J Mol Sci. (2020) 21:3422. doi: 10.3390/ijms21103422 32408613 PMC7279398

[28] Lagnese G Messuri C Poto R Varricchi G Spadaro G . Secondary antibody deficiencies: What’s around the corner? Front Immunol. (2025) 16. doi: 10.3389/fimmu.2025.1672413 41425555 PMC12711868

[29] Science M Akseer N Asner S Allen U . Risk stratification of immunocompromised children, including pediatric transplant recipients at risk of severe respiratory syncytial virus disease. Pediatr Transplant. (2019) 23:e13336. doi: 10.1111/petr.13336 30604582

[30] Schäfer A Kovacs MS Eder A Nigg A Feuchtenberger M . Janus kinase (JAK) inhibitors significantly reduce the humoral vaccination response against SARS-CoV-2 in patients with rheumatoid arthritis. Clin Rheumatol. (2022) 41:3707–14. doi: 10.1007/s10067-022-06329-2 35965290 PMC9376125

[31] Santos DMCD Tix T Shouval R Gafter-Gvili A Alberge J Cliff ERS . A systematic review and meta-analysis of nonrelapse mortality after CAR T cell therapy. Nat Med. (2024) 30:2667–78. doi: 10.1038/s41591-024-03084-6 38977912 PMC11765209

[32] Ross JB Myers LM Noh JJ Collins MM Carmody AB Messer RJ . Depleting myeloid-biased hematopoietic stem cells rejuvenates aged immunity. Nature. (2024). doi: 10.1038/s41586-024-07238-x 38538791 PMC11870232

[33] Nass N Yaakoub MK Simion AV Kroll H Westphal S Pannier J . SARS-CoV-2 vaccine-induced seroconversion and immune correlates in patients with hematological Malignancies. A real world study. Oncol Res Featuring Preclinical Clin Cancer Ther. (2025). doi: 10.32604/or.2025.067561 41050077 PMC12494110

[34] Ridker PM Everett BM Thuren T MacFadyen JG Chang WH Ballantyne C . Anti-inflammatory therapy with canakinumab for atherosclerotic disease. N Engl J Med. (2017). doi: 10.1056/nejmoa1707914 28845751

[35] Reynolds G Cliff ERS Mohyuddin GR Popat R Midha S Hing MNL . Infections following bispecific antibodies in myeloma: a systematic review and meta-analysis. Blood Adv. (2023) 7:5898–903. doi: 10.1182/bloodadvances.2023010539 37467036 PMC10558589

[36] Regina J Doms J Kampouri E Gerber C Manuel O Bart PA . Immunodeficiencies in adults: key considerations for diagnosis and management. Clin Rev Allergy Immunol. (2025) 68:92. doi: 10.1007/s12016-025-09103-9 41107625 PMC12534305

[37] MaChado CM . Reimmunization after hematopoietic stem cell transplantation. Expert Rev Vaccines. (2005) 4:219–28. doi: 10.1586/14760584.4.2.219 15889995

[38] Balmaceda N Aziz M Chandrasekar VT McClune B Kambhampati S Shune L . Infection risks in multiple myeloma: A systematic review and meta-analysis of randomized trials from 2015 to 2019. BMC Cancer. (2021) 21:730. doi: 10.1186/s12885-021-08451-x 34172037 PMC8233183

[39] Ladomenou F Gaspar B . How to use immunoglobulin levels in investigating immune deficiencies. Arch Dis Childhood Educ Pract. (2016) 101:129–35. doi: 10.1136/archdischild-2015-309060 26987724

[40] Plonsky-Toder M Magen D Pollack S . Innate immunity and CKD: Is there a significant association? Cells. (2023) 12(23):235–52. doi: 10.3390/cells12232714 PMC10705738 38067142

[41] Pich LMM Ziogas A Netea MG . Genetic and epigenetic dysregulation of innate immune mechanisms in autoinflammatory diseases. FEBS J. (2024) 291:4414–32. doi: 10.1111/febs.17116 38468589

[42] Peter JG Heckmann JM Novitzky N . Recommendations for the use of immunoglobulin therapy for immunomodulation and antibody replacement. South Afr Med J. (2014) 104:796. doi: 10.7196/samj.8965 29183440

[43] Peng JM Du B Qin HY Wang Q Shi Y . Metagenomic next-generation sequencing for the diagnosis of suspected pneumonia in immunocompromised patients. J Infect. (2021) 82:22–7. doi: 10.1016/j.jinf.2021.01.029 33609588

[44] Paulsen JS Slørdahl TS . To substitute or not? A systematic review of immunoglobulin replacement therapy in multiple myeloma patients treated with bispecific antibodies. Front Immunol. (2026). doi: 10.3389/fimmu.2025.1722579 41601684 PMC12832799

[45] Patel SY Carbone J Jolles S . The expanding field of secondary antibody deficiency: causes, diagnosis, and management. Front Immunol. (2019) 10. doi: 10.3389/fimmu.2019.00033 30800120 PMC6376447

[46] Parikh SA Leis JF Chaffee KG Call TG Hanson CA Ding W . Hypogammaglobulinemia in newly diagnosed chronic lymphocytic leukemia: natural history, clinical correlates, and outcomes. Cancer. (2015) 121:2883–91. doi: 10.1002/cncr.29438 PMC4545721 25931291

[47] Paiardini M Müller‐Trutwin M . HIV‐associated chronic immune activation. Immunol Rev. (2013). doi: 10.1111/imr.12079 23772616 PMC3729961

[48] Page MJ McKenzie JE Bossuyt PM Boutron I Hoffmann TC Mulrow CD . The PRISMA 2020 statement: an updated guideline for reporting systematic reviews. BMJ. (2021) 372:n71. doi: 10.1136/bmj.n71 33782057 PMC8005924

[49] Padurariu-Covit MD Georgescu C Andreescu M Chiscop I Plesea-Condratovici C Arbune M . CAR-T cell therapy for HIV cure: current challenges, advances, and future directions. Viruses. (2025) 17:1615. doi: 10.3390/v17121615 41472284 PMC12737754

[50] Otani IM Lehman HK Jongco AM Tsao LR Azar AE Tarrant TK . Practical guidance for the diagnosis and management of secondary hypogammaglobulinemia: a work group report of the AAAAI Primary Immunodeficiency and Altered Immune Response Committees. J Allergy Clin Immunol. (2022) 149:1525–60. doi: 10.1016/j.jaci.2022.01.025 35176351

[51] Ohkuma R Onishi N Watanabe M Jin J Ariizumi H Shimokawa M . Elevated plasma IgG is associated with improved treatment response and survival in advanced non-small cell lung cancer patients treated with anti-PD1 therapy. Nature. (2026). doi: 10.1038/s41598-026-46923-x 42009794 PMC13260361

[52] O’Donnell E Gift T Yousif Z Khandelwal N Desai R Huynh L . Real-world effectiveness of immunoglobulin replacement for hypogammaglobulinemia and infections in multiple myeloma. Blood Adv. (2025) 9:3780–9. doi: 10.1182/bloodadvances.2024015477 40305667 PMC12309603

[53] Neofytos D Steinbach WJ Hanson K Carpenter PA Papanicolaou GA Slavin MA . American Society for Transplantation and Cellular Therapy Series, #6: Management of invasive candidiasis in hematopoietic cell transplantation recipients. Transplant Cell Ther. (2023) 29:222–7. doi: 10.1016/j.jtct.2023.01.011 36649748

[54] Na I Buckland M Agostini C Edgar JDM Friman V Michallet M . Current clinical practice and challenges in the management of secondary immunodeficiency in hematological Malignancies. Eur J Hematol. (2019) 102:447–56. doi: 10.1111/ejh.13223 30801785 PMC6849602

[55] Mears V Jakubecz C Seeco C Woodson S Serra A Abboud H . Predictors of hypogammaglobulinemia and serious infections among patients receiving ocrelizumab or rituximab for treatment of MS and NMOSD. J Neuroimmunol. (2023). doi: 10.1016/j.jneuroim.2023.578066 36917920

[56] Hallberg S Evertsson B Lillvall E Boremalm M Flon P Wang Y . Hypogammaglobulinemia during rituximab treatment in multiple sclerosis: A Swedish cohort study. Eur J Neurol. (2024). doi: 10.1111/ene.16331 38794973 PMC11236063

[57] McDonagh TA Metra M Adamo M Gardner RS Baumbach A Böhm M . ESC guidelines for the diagnosis and treatment of acute and chronic heart failure. Eur Heart J. (2021) 42:3599–726. doi: 10.1093/eurheartj/ehab368 34922348

[58] Mazzuti L Turriziani O Mezzaroma I . The many faces of immune activation in HIV-1 infection: a multifactorial interconnection. Biomedicines. (2023) 11:159. doi: 10.3390/biomedicines11010159 36672667 PMC9856151

[59] Maeda Y Caskurlu S Kenney RH Kozan K Richardson JC . Moving qualitative synthesis research forward in education: a methodological systematic review. Educ Res Rev. (2021) 35:100424. doi: 10.1016/j.edurev.2021.100424 38826717

[60] Long A Kleiner A Looney RJ . Immune dysregulation. J Allergy Clin Immunol. (2023). doi: 10.1016/j.jaci.2022.11.001 36608984

[61] Lin JS Evans CV Grossman DC Tseng CW Krist AH . Framework for using risk stratification to improve clinical preventive service guidelines. Am J Prev Med. (2017) 54:S26–37. doi: 10.1016/j.amepre.2017.07.023 29254523

[62] Lauxen JS Vondenhoff S Junho CVC Martin P Schütt K . Neutrophil function in patients with chronic kidney disease: A systematic review and meta‐analysis. Acta Physiologica (2025). 10.1111/apha.70057 PMC12102643 40411205

[63] Kumar M Yip L Wang F Marty SE Fathman CG . Autoimmune disease: Genetic susceptibility, environmental triggers, and immune dysregulation. Where can we develop therapies. Front Immunol. (2025). 10.3389/fimmu.2025.1626082 PMC12367657 40852720

[64] Kinyoki D Osgood-Zimmerman AE Bhattacharjee NV DA CLB Schaeffer LE Lazzar-Atwood A . Anemia prevalence in women of reproductive age in low- and middle-income countries between 2000 and 2018. Nat Med. (2021) 27:1761–82. doi: 10.1038/s41591-021-01498-0 34642490 PMC8516651

[65] Elkhalifa S Salvo F Elbashir H Shafiq I Isse S Abuzakouk M . Secondary antibody deficiencies in the modern era: Emerging trends, diagnostic pitfalls, and advances in personalized management. Front Immunol. (2025). 10.3389/fimmu.2025.1635094 PMC12537668 41126828

[66] Eibl MM Wolf HM . Vaccination in patients with primary immune deficiency, secondary immune deficiency, and autoimmunity with immune regulatory abnormalities. Immunotherapy. (2015) 7:1273–92. doi: 10.2217/imt.15.74 26289364

[67] Khan SR Chaker L Ikram MA Peeters RP van Hagen PM Dalm VASH . Determinants and reference ranges of serum immunoglobulins in middle-aged and elderly individuals: A population-based study. J Clin Immunol. (2021) 41:1902–14. doi: 10.1007/s10875-021-01120-5 34505230 PMC8604889

[68] Kan AKC Chan BCY Lau CS Li PH . Bridging the gap in secondary antibody deficiencies: Current evidence and unmet needs in diagnosis and management with immunoglobulin replacement. Clin Rev Allergy Immunol. (2025) 68:106. doi: 10.1007/s12016-025-09116-4 41351689 PMC12681477

[69] Jolles S Giralt S Kerre T Lazarus HM Mustafa SS Ria R . Agents contributing to secondary immunodeficiency development in patients with multiple myeloma, chronic lymphocytic leukemia, and non-Hodgkin lymphoma: A systematic literature review. Front Oncol. (2023) 13. doi: 10.3389/fonc.2023.1098326 36824125 PMC9941665

[70] Jolles S Giralt S Kerre T Lazarus HM Mustafa SS Papanicolaou GA . Secondary antibody deficiency in chronic lymphocytic leukemia and non-Hodgkin lymphoma: Recommendations from an international expert panel. Blood Rev. (2022) 58:101020. doi: 10.1016/j.blre.2022.101020 38826717

[71] Irvine KM Ratnasekera I Powell EE Hume DA . Causes and consequences of innate immune dysfunction in cirrhosis. Front Immunol. (2019). doi: 10.3389/fimmu.2019.00293 30873165 PMC6401613

[72] Holub M Cheng CW Mott S Wintermeyer P Rooijen N Gregory SH . Neutrophils sequestered in the liver suppress the proinflammatory response of Kupffer cells to systemic bacterial infection. J Immunol. (2009). doi: 10.4049/jimmunol.0803041 19641138

[73] Hatcher VR Alix VC Hellu TS Schuldt MM . Primary immunodeficiency: Specific antibody deficiency with normal IgG. Allergy Asthma Proc. (2024) 45:321–5. doi: 10.2500/aap.2024.45.240057 39294904 PMC11441535

[74] Gualtieri R Yerly S Garcia‐Tarodo S Parvex P Rock N Posfay‐Barbe K . Rituximab‐to‐vaccine interval on SARS‐CoV‐2 immunogenicity in children: The potential role of prior natural infection. Pediatr Allergy Immunol. (2024). doi: 10.1111/pai.14161 38796784

[75] Grigoriadou S Clubbe R Garcez T Huissoon A Grosse-Kreul D Jolles S . British Society for Immunology and United Kingdom Primary Immunodeficiency Network (UKPIN) consensus guideline for the management of immunoglobulin replacement therapy. Clin Exp Immunol. (2022). 10.1093/cei/uxac070 PMC9585546 35924867

[76] Goyani P Christodoulou R Vassiliou E . Immunosenescence: Aging and immune system decline. Vaccines. (2024) 12:1314. doi: 10.3390/vaccines12121314 39771976 PMC11680340

[77] Dagnew AF Ilhan O Lee WS Woszczyk D Kwak JY Bowcock S . 149. Immunogenicity, safety, and post-hoc efficacy assessment of the adjuvanted recombinant zoster vaccine in adults with hematologic Malignancies: A phase 3, randomized clinical trial. Open Forum Infect Dis. (2018). doi: 10.1093/ofid/ofy209.019 31399377

[78] Burger JA . Bruton tyrosine kinase inhibitors. Cancer J. (2019) 25:386–93. doi: 10.1097/ppo.0000000000000412 31764119 PMC7083517

[79] Baral S . Implementing resource-stratified guidelines in LMICs: More issues than solutions. JAMA Oncol. (2024) 10:1613. doi: 10.1001/jamaoncol.2024.4351 39388172

[80] van Kessel DA Hoffman TW van Velzen-Blad H Zanen P Grutters JC Rijkers GT . Long-term clinical outcome of antibody replacement therapy in humoral immunodeficient adults with respiratory tract infections. EBioMedicine. (2017). doi: 10.1016/j.ebiom.2017.03.025 28347655 PMC5405168

[81] Giannella M Riccucci D Pascale R Cordero E Mueller NJ Slavin M . Current practice patterns and educational needs of the ESCMID Study Group for infections in compromised hots. Transplant Infect Dis. (2025) 27:e70076. doi: 10.1111/tid.70076 40625111 PMC12416473

[82] Chai KL Wood EM Estcourt LJ Csenar M Iannizzi C Monsef I . Immunoglobulin replacement to prevent infections in people with hematological Malignancies and hematopoietic stem cell transplantation. Cochrane Database Syst Rev. (2024).

[83] Chai KL Wong J Weinkove R Keegan A Crispin P Stanworth S . Interventions to reduce infections in patients with hematological Malignancies: A systematic review and meta-analysis. Blood Adv. (2022) 7:20–31. doi: 10.1182/bloodadvances.2022008073 35882473 PMC9813525

[84] Gerek ME Tekinalp A Çölkesen F Önalan T Akkuş FA Kılınç M . Pretreatment immunoglobulin profiles and survival outcomes in hematologic Malignancies and autoimmune cytopenias: A comparative cohort analysis. BMC Immunol. (2026). doi: 10.1186/s12865-026-00813-z 41673825 PMC12997871

[85] Galaviz KI Gonzalez-Casanova I Alonso A . Risk prediction in people living with human immunodeficiency virus: Are we hitting the target. Clin Infect Dis. (2019). 10.1093/cid/ciz1219 PMC7947977 31909788

[86] Bonet CM Waser N Cheng K Tzivelekis S Edgar JDM Sánchez-Ramón S . A systematic literature review of the effects of immunoglobulin replacement therapy on the burden of secondary immunodeficiency diseases associated with hematological Malignancies and stem cell transplants. Expert Rev Clin Immunol. (2020) 16:911–21. doi: 10.1080/1744666x.2020.1807328 32783541

[87] Friman V Winqvist O Blimark C Langerbeins P Chapel H Dhalla F . Secondary immunodeficiency in lymphoproliferative Malignancies. Hematol Oncol. (2016) 34:121–32. doi: 10.1002/hon.2323 27402426

[88] Ecarnot F Maggi S . Vaccination against respiratory infections in the immunosenescent older adult population: Challenges and opportunities. Semin Respir Crit Care Med. (2024) 46:053–62. doi: 10.1055/a-2500-2121 39662893

[89] Duraisingham SS Buckland MS Grigoriadou S Longhurst HJ . Secondary antibody deficiency. Expert Rev Clin Immunol. (2014) 10:583–91. doi: 10.1586/1744666x.2014.902314 24684706

[90] Du FH Mills EA Mao-Draayer Y . Next-generation anti-CD20 monoclonal antibodies in autoimmune disease treatment. Autoimmun Highlights. (2017) 8:12. doi: 10.1007/s13317-017-0100-y 29143151 PMC5688039

[91] Banerjee R Mohan M Rejeski K Puliafito BR Cirstea DD Kaur G . Immunoglobulin prophylaxis should be initiated after bispecific antibody therapy in multiple myeloma, regardless of IgG levels. Blood Adv. (2025) 9:4720–6. doi: 10.1182/bloodadvances.2025016490 40690761 PMC12466225

[92] Chen Y Chen Z Cao J Lin L Li J . Severe and persistent immunoparesis during induction or maintenance therapy in non-transplant patients with multiple myeloma is a poor prognostic sign. Hematology. (2024) 29:2329378. doi: 10.1080/16078454.2024.2329378 38470208

[93] Bayly-McCredie E Treisman M Fiorenza S . Safety and efficacy of bispecific antibodies in adults with large B-cell lymphomas: A systematic review of clinical trial data. Int J Mol Sci. (2024) 25:9736. doi: 10.3390/ijms25179736 39273684 PMC11396745

[94] Ball S Das A Vutthikraivit W Edwards PJ Hardwicke F Short NJ . Risk of infection associated with ibrutinib in patients with B-cell Malignancies: A systematic review and meta-analysis of randomized controlled trials. Clin Lymphoma Myeloma Leuk. (2019) 20:5. doi: 10.1016/j.clml.2019.10.004 31787589

[95] Allegra A Tonacci A Musolino C Pioggia G Gangemi S . Secondary immunodeficiency in hematological Malignancies: Focus on multiple myeloma and chronic lymphocytic leukemia. Front Immunol. (2021) 12. doi: 10.3389/fimmu.2021.738915 34759921 PMC8573331

[96] Aktaş ÖÖ Özer NO Kaplankıran C Akın BG Ozturk BO Durmaz MSB . Immunoglobulin G trough levels and infection risk in adults with inborn errors of immunity receiving immunoglobulin therapy. Medicina. (2025) 61:1549. doi: 10.3390/medicina61091549 41010940 PMC12471541

[97] Agrawal S Bapat A Amos J Howes E Ashfield T . Adopting prospective antimicrobial stewardship (AMS) practice in high-risk immunosuppressed groups: An urgent call to action in the era of antimicrobial resistance (AMR. JAC Antimicrob Resist. (2024) 6:dlae145. doi: 10.1093/jacamr/dlae145 39346971 PMC11428039

[98] Agostini C Blau I Kimby E Plesner T . Prophylactic immunoglobulin therapy in secondary immune deficiency – An expert opinion. Expert Rev Clin Immunol. (2016) 12:921–6. doi: 10.1080/1744666x.2016.1208085 27415820

[99] Aalst M Langedijk AC Spijker R Bree GJ Grobusch MP Goorhuis A . The effect of immunosuppressive agents on immunogenicity of pneumococcal vaccination: A systematic review and meta-analysis. Vaccine. (2018) 36:5832–45. doi: 10.1016/j.vaccine.2018.07.039 30122649

[100] Ballow M Sánchez-Ramón S Walter JE . Secondary immune deficiency and primary immune deficiency crossovers: hematological malignancies and autoimmune diseases. Front. Immunol. (2022) 13:928062. doi: 10.3389/fimmu.2022.928062 35924244 PMC9340211

