# Supplementary material for: Rethinking secondary immunodeficiency: a cross-domain pathway framework for risk stratification
Source: Front Immunol. 2026 Aug 26;17:1866662. doi: 10.3389/fimmu.2026.1866662 (PMC13553538; doi:10.3389/fimmu.2026.1866662)
Supplement: Supplementary file 1 [file SupplementaryFile1.docx]

Supplementary Material

# 1 Supplementary Data

# 2  Appendix A: Detailed Review Protocol

**Protocol for a Systematic Review: Rethinking Secondary Immunodeficiency: A Cross-Domain Pathway Framework for Risk Stratification**

**1. Background and Rationale**

Secondary immunodeficiency (SID) is an acquired impairment of immune function arising from malignancy, immunomodulatory therapies, chronic infection, organ dysfunction, malnutrition, and aging. Unlike primary immunodeficiencies, SID reflects dynamic and exposure-driven immune disruption, often evolving over time and across clinical contexts.

Despite increasing global prevalence, SID continues to be conceptualized within disease-specific silos. Hematology, rheumatology, infectious disease, and transplant medicine each apply distinct diagnostic thresholds, immune monitoring strategies, and treatment approaches. This fragmentation results in inconsistent identification of clinically meaningful immune dysfunction and variable access to preventive interventions such as immunoglobulin replacement therapy (IgRT), vaccination, and antimicrobial prophylaxis.

Emerging evidence suggests that, irrespective of underlying etiology, SID converges into three reproducible immunological pathways:

- Humoral dysfunction
- Cellular disruption
- Innate immune dysregulation

However, no prior systematic synthesis has integrated these mechanistic pathways with diagnostic heterogeneity and clinical management across domains. Existing reviews remain limited by disease-specific scope and lack of translational frameworks.

This protocol outlines a systematic review using qualitative thematic synthesis and cross-domain mapping to:

1. Identify convergent immunological mechanisms across SID etiologies
2. Examine inconsistencies in diagnostic and management approaches
3. Develop an exploratory, pathway-based risk stratification framework

The review is conducted in accordance with PRISMA 2020.

**2. Objectives**

**Primary Objective**

To synthesize evidence on immunological mechanisms, diagnostic practices, infection patterns, and management strategies across secondary immunodeficiency, and to identify cross-domain convergence using a pathway-based analytical model.

**Secondary Objectives**

1. To map SID-related findings onto three predefined immunological pathways: humoral, cellular, and innate
2. To evaluate variability in diagnostic criteria, including immunoglobulin thresholds and functional immune assessment
3. To examine associations between immune dysfunction and infection outcomes
4. To assess evidence supporting management strategies (IgRT, vaccination, antimicrobial prophylaxis, and immune monitoring)
5. To develop an exploratory cross-domain SID Risk Stratification Framework based on convergent evidence
6. To identify global applicability and gaps, including relevance to low- and middle-income settings

**3. Eligibility Criteria (PICOS Framework)**

**Population**

Adults and children with secondary immunodeficiency arising from acquired conditions, including:

- Hematological malignancies
- Autoimmune/biologic therapies
- Transplantation (SOT and HSCT) / CKD and organ failure
- HIV/malnutrition/Aging
- Immunosenescence

Primary immunodeficiency studies will be excluded unless clearly separable.

**Intervention/Exposure**

- Underlying diseases or conditions causing SID
- Immunosuppressive or immunomodulatory therapies
- Transplant-related immune suppression
- Nutritional or physiological immune impairment

**Comparator**

Where applicable:

- Non-SID populations
- Alternative exposures or treatment strategies
- Pre–post comparisons

**Outcomes**

***Primary Outcomes***

- Infection incidence and severity
- Infection-related hospitalization
- Infection-related mortality

***Secondary Outcomes***

- Immunoglobulin levels
- Functional antibody responses
- Lymphocyte subsets and immune markers
- Vaccine response
- Clinical management outcomes

**Study Design**

**Included:**

- 20 Randomized Controlled Trials
- 48 Observational Studies
- 12 Registry-Based Studies
- 20 Systematic Reviews

**Excluded:**

- Case reports or small case series
- Editorials and commentaries
- Conference abstracts without full text
- Non-human studies

**Restrictions:**

- English language
- Publication period: January 2005 to March 2026.

**4. Databases and Search Strategy**

Databases to be searched:

- PubMed/MEDLINE
- Embase
- Scopus
- Web of Science

Additional sources:

- Reference list screening
- Forward citation tracking

**Search Strategy**

Search terms will combine controlled vocabulary and free-text terms, including:

- “Secondary immunodeficiency”
- “hypogammaglobulinemia”
- “Immune dysfunction”
- “Infection susceptibility”
- “Immunosuppressive therapy”
- “transplantation”
- “HIV”
- “malnutrition”
- “immunosenescence”

A full reproducible strategy will be provided in the supplementary material.

**5. Screening Process**

A two-stage screening process will be applied:

**Stage 1: Title and Abstract Screening**

- Conducted by the primary reviewer
- Calibration performed on a random subset
- Agreement assessed (Cohen’s κ ≥ 0.80)

**Stage 2: Full-Text Review**

- Eligibility assessed using predefined criteria
- Discrepancies resolved through discussion or adjudication

A PRISMA flow diagram will document the selection.

**6. Data Extraction Plan**

A standardized, piloted extraction form will be used.

**Extracted Variables**

- Study characteristics
- Population and clinical domain
- SID definition and diagnostic criteria
- Immunological findings
- Infection outcomes
- Management strategies
- Key conclusions

A subset (≈20%) will undergo independent verification.

**7. Risk of Bias Assessment**

Design-specific tools will be used:

- **RoB 2** (randomized trials)
- **Newcastle–Ottawa Scale (NOS)** (observational studies)
- **AMSTAR-2** (systematic reviews)

Assessment will be conducted independently by two reviewers.
Findings will inform interpretation, not exclusion.

**8. Synthesis Plan**

Given expected heterogeneity, **meta-analysis will not be conducted**.

Instead, a structured qualitative synthesis will be applied:

**Three-Stage Approach**

1. Line-by-line coding of extracted findings
2. Axial aggregation into themes
3. Cross-domain synthesis using predefined pathways

**Analytical Framework**

All findings will be mapped onto three axes:

- Humoral dysfunction
- Cellular disruption
- Innate immune dysregulation

**Output**

- Domain-level synthesis
- Cross-domain comparisons
- Descriptive quantitative summaries (frequencies, ranges)

**9. Framework Development Methodology**

An exploratory SID Risk Stratification Framework will be developed using a **convergence-based approach:**

### 2.1.1 **Inclusion Criteria for Variables**

A variable must:

- Appear in ≥10% of studies OR
- Be reported across ≥3 clinical domains

AND

- Show consistent association with clinical outcomes

### 2.1.2 **Structure**

Variables will be classified into:

- Immunological markers
- Clinical outcomes
- Therapeutic exposures
- Host factors

### 2.1.3 **Weighting**

Assigned qualitatively based on:

- Frequency of reporting
- Consistency of association
- Clinical relevance

### 2.1.4 **Nature of Framework**

- Conceptual and exploratory; synthesizes convergent evidence into a structured clinical reasoning tool
- Not statistically validated or prospectively tested
- Intended for future prospective validation across disease domains, age groups, and health system contexts

**10. Certainty Assessment Plan**

Certainty of evidence will be evaluated qualitatively using **GRADE-informed principles**, considering:

- Risk of bias
- Consistency
- Directness
- Completeness

Formal GRADE tables may be generated where feasible.

**11. Timeline**

- Protocol development and registration: Completed
- Literature search: Month 1
- Screening: Months 1–2
- Data extraction and bias assessment: Months 2–3
- Synthesis and framework development: Months 3–4
- Manuscript finalization: Month 5

**12. Amendments Policy**

Any deviations from the protocol will be:

- Documented with date and rationale
- Reported transparently in the final manuscript

No post hoc outcome modification will be permitted.

# 3 Appendix B: Supplementary Tables

| Supplementary Table S1: Online Repository (Study-Level Summary) | | | | | | | | | | |
| --- | --- | --- | --- | --- | --- | --- | --- | --- | --- | --- |
| ID | | **First Author & Year** | | **Study Type** | | **Population/Condition** | | **SID Cause/Focus** | | **Key Focus/Outcome** |
| 1 | Allegra 2021 | | Systematic Review | | Multiple myeloma, CLL | | Disease-related SID | | Mechanisms and clinical burden of secondary immunodeficiency in hematologic malignancies | |
| 2 | Ball 2019 | | Systematic Review & Meta-analysis | | B-cell malignancies (treated with ibrutinib) | | BTK inhibitor-induced immunosuppression | | Increased infection risk associated with ibrutinib therapy | |
| 3 | Balmaceda 2021 | | Systematic Review & Meta-analysis | | Multiple myeloma patients | | Treatment-related immunosuppression | | Quantified infection risk and incidence in MM clinical trials | |
| 4 | Banerjee 2025 | | Observational Study | | Multiple myeloma (bispecific antibody therapy) | | Therapy-induced hypogammaglobulinemia | | Supports early immunoglobulin prophylaxis regardless of IgG levels | |
| 5 | Wijetilleka 2019 | | Systematic Review | | Autoimmune rheumatic diseases | | B-cell targeted therapy (e.g., rituximab) | | Evaluates the effectiveness of immunoglobulin replacement therapy (IgRT) | |
| 6 | Elkhalifa 2025 | | Systematic Review | | Broad SID populations | | Multiple causes (therapy, disease) | | Emerging trends, diagnostic challenges, and personalized management of antibody deficiency | |
| 7 | Chai 2022 | | Systematic Review & Meta-analysis | | Hematologic malignancies | | Infection prevention strategies | | Effectiveness of interventions in reducing infection risk | |
| 8 | Christou 2017 | | Systematic Review | | Rituximab-treated patients | | Anti-CD20 therapy | | Risk factors for hypogammaglobulinemia and infections | |
| 9 | Lagnese 2025 | | Systematic Review | | Secondary antibody deficiency | | General SID mechanisms | | Future perspectives and evolving understanding of antibody deficiencies | |
| 10 | Friman 2016 | | Systematic Review | | Lymphoproliferative malignancies | | Disease- and therapy-induced SID | | Clinical implications and management challenges of SID | |
| 11 | Na 2019 | | Observational Study | | Hematologic malignancies | | SID management strategies | | Gaps in clinical practice and real-world management challenges | |
| 12 | Page 2021 | | Guideline/Reporting Standard | | Systematic reviews (methodology) | | Not disease-specific | | PRISMA 2020 framework for transparent reporting | |
| 13 | Jolles 2023 | | Systematic Review | | Multiple myeloma, CLL, NHL | | Therapy-induced SID (various agents) | | Identifies drugs contributing to secondary immunodeficiency development | |
| 14 | Reynolds 2023 | | Systematic Review & Meta-analysis | | Multiple myeloma (bispecific antibody therapy) | | Treatment-related infections | | Quantifies infection incidence following bispecific antibody use | |
| 15 | Santos 2024 | | Systematic Review & Meta-analysis | | Patients undergoing CAR-T therapy | | Treatment-related mortality | | Evaluates non-relapse mortality and associated risks post CAR-T | |
| 16 | Aktaş 2025 | | Observational Study | | Adults with inborn errors of immunity on IgRT | | IgG trough levels | | Correlates IgG levels with infection risk | |
| 17 | Vijenthira 2021 | | Systematic Review & Meta-analysis | | Patients receiving anti-CD20 therapy | | Impaired vaccine response | | Showed reduced vaccine immunogenicity | |
| 18 | Aalst 2018 | | Systematic Review & Meta-analysis | | Immunocompromised patients | | Immunosuppressive drugs | | Impact of therapies on pneumococcal vaccine response | |
| 19 | Maeda 2021 | | Methodological Systematic Review | | Educational research studies | | Research synthesis methods | | Advances in the methodology of qualitative systematic reviews | |
| 20 | Sterne 2019 | | Methodological Guideline | | Randomized trials | | Risk of bias assessment | | Introduces the RoB 2 tool for bias evaluation | |
| 21 | Stang 2010 | | Methodological Review | | Observational studies | | Quality assessment tools | | Critiques Newcastle-Ottawa Scale limitations | |
| 22 | Shea 2017 | | Methodological Guideline | | Systematic reviews | | Quality appraisal | | Introduces AMSTAR 2 for evaluating systematic reviews | |
| 23 | Bayly-McCredie 2024 | | Systematic Review | | Large B-cell lymphoma | | Bispecific antibody safety | | Assesses safety and efficacy outcomes | |
| 24 | Yeung 2024 | | Systematic Review | | Rheumatic diseases | | Hypogammaglobulinemia monitoring | | Highlights the importance of Ig monitoring in SID | |
| 25 | Bonet 2020 | | Systematic Review | | Hematologic malignancies, HSCT | | IgRT effectiveness | | Evaluates burden reduction with immunoglobulin therapy | |
| 26 | Kan 2025 | | Systematic Review | | Secondary antibody deficiency (various conditions) | | Diagnostic and therapeutic gaps | | Highlights unmet needs in diagnosis and IgRT management | |
| 27 | Neofytos 2023 | | Clinical Guideline/Review | | Hematopoietic cell transplant recipients | | Opportunistic infections (fungal) | | Evidence-based management of invasive candidiasis | |
| 28 | Ueda 2017 | | Systematic Review | | Hematologic malignancies and HSCT | | Hypogammaglobulinemia | | Role of immunoglobulin therapy in the prevention of infections | |
| 29 | Hatcher 2024 | | Systematic Review | | Primary immunodeficiency (SAD with normal IgG) | | Antibody dysfunction | | Clinical characterization of specific antibody deficiency | |
| 30 | Agostini 2016 | | Registry-Based Studies | | Secondary immunodeficiency | | IgRT use | | Recommends prophylactic immunoglobulin therapy in SID | |
| 31 | Jolles 2022 | | Registry-Based Studies | | CLL and NHL | | Secondary antibody deficiency | | Provides recommendations for diagnosis and management | |
| 32 | Parikh 2015 | | Observational Cohort Study | | Newly diagnosed CLL patients | | Disease-related hypogammaglobulinemia | | Natural history and prognostic implications of low Ig levels | |
| 33 | Chen 2024 | | Observational Study | | Multiple myeloma (non-transplant) | | Immunoparesis during therapy | | Persistent immunosuppression is linked to poor prognosis | |
| 34 | Burger 2019 | | Systematic Review | | B-cell malignancies | | BTK inhibitors | | Mechanism and clinical use of BTK inhibitors | |
| 35 | Paulsen 2026 | | Systematic Review | | Multiple myeloma (bispecific antibody therapy) | | IgRT in SID | | Evaluates the need for immunoglobulin replacement in treated MM | |
| 36 | Sutherland 2024 | | Observational Study | | CAR-T cell therapy recipients | | Hypogammaglobulinemia and infections | | Links CAR-T therapy with infection risk and mortality | |
| 37 | Nass 2025 | | Observational Study | | Hematologic malignancies | | SARS-CoV-2 vaccine response | | Reduced seroconversion in immunocompromised patients | |
| 38 | Grigoriadou 2022 | | Clinical Guideline | | Primary & secondary immunodeficiency | | IgRT management | | Standardizes immunoglobulin replacement practices | |
| 39 | Tallantyre 2018 | | Observational Study | | Neuroinflammatory diseases (anti-CD20 therapy) | | Secondary antibody deficiency | | Identifies SID as a complication of anti-CD20 therapy | |
| 40 | Hallberg 2024 | | Observational Cohort Study | | Multiple sclerosis (rituximab-treated) | | Hypogammaglobulinemia | | Long-term risk of low Ig levels during rituximab treatment | |
| 41 | Mears 2023 | | Observational Study | | MS and NMOSD patients (rituximab/ocrelizumab) | | Anti-CD20 therapy | | Identifies predictors of hypogammaglobulinemia and serious infections | |
| 42 | Schäfer 2022 | | Observational Study | | Rheumatoid arthritis patients | | JAK inhibitor therapy | | Reduced humoral response to SARS-CoV-2 vaccination | |
| 43 | Dimou 2025 | | Systematic Review | | Secondary immunodeficiency patients | | IgRT (subcutaneous) | | Effectiveness, safety, and tolerability of Ig replacement therapy | |
| 44 | Du 2017 | | Systematic Review | | Autoimmune diseases | | Anti-CD20 monoclonal antibodies | | Mechanisms and clinical applications of next-generation therapies | |
| 45 | Eibl 2015 | | Systematic Review | | PID, SID, autoimmune disorders | | Vaccination in immunodeficiency | | Vaccination strategies in immunocompromised populations | |
| 46 | Machado 2005 | | Systematic Review | | HSCT recipients | | Post-transplant immune recovery | | Reimmunization strategies after transplantation | |
| 47 | Syed-Ahmed 2019 | | Systematic Review | | Chronic kidney disease (CKD) | | Immune dysfunction | | Increased infection susceptibility in CKD | |
| 48 | Viasus 2011 | | Observational Study | | CKD patients with pneumonia | | Comorbidity-related immune dysfunction | | Clinical outcomes and severity of infections in CKD | |
| 49 | Holub 2009 | | Experimental Study | | Animal model (murine infection) | | Innate immune regulation | | Neutrophil-mediated suppression of inflammatory response | |
| 50 | Mazzuti 2023 | | Systematic Review | | HIV-1 infection | | Chronic immune activation | | Multifactorial drivers of immune activation in HIV | |
| 51 | Paiardini 2013 | | Systematic Review | | HIV infection | | Chronic immune activation | | Mechanisms linking immune activation to disease progression | |
| 52 | Shao 2021 | | Systematic Review | | General population | | Lifestyle and immune modulation | | Role of nutrition and physical activity in immune function | |
| 53 | Ross 2024 | | Experimental Study | | Aging immune system (murine model) | | Hematopoietic stem cell aging | | Rejuvenation of immune function via stem cell targeting | |
| 54 | Goyani 2024 | | Systematic Review | | Elderly population | | Immunosenescence | | Age-related immune decline and vaccine implications | |
| 55 | Thomas 2020 | | Systematic Review | | Aging population | | Thymic involution | | Contribution of thymic decline to immunosenescence | |
| 56 | Ecarnot 2024 | | Systematic Review | | Elderly population | | Immunosenescence | | Vaccination challenges and opportunities in aging immune systems | |
| 57 | Khan 2021 | | Observational Study | | Middle-aged and elderly individuals | | Baseline immunoglobulin variation | | Defines reference ranges for serum immunoglobulins | |
| 58 | Ladomenou 2016 | | Clinical Review | | Suspected immunodeficiency patients | | Diagnostic use of Ig levels | | Practical interpretation of immunoglobulin levels in diagnosis | |
| 59 | Torab-Miandoab 2024 | | Methodological/Technical Study | | Healthcare systems | | Data interoperability | | Proposes a data-driven framework for healthcare integration | |
| 60 | Shi 2020 | | Methodological Review | | Immune system research | | Systems immunology | | Network-based approaches to studying immune interactions | |
| 61 | Lin 2017 | | Conceptual/Methodological Framework | | General population | | Risk stratification | | Framework for improving preventive care using risk stratification | |
| 62 | Otani 2022 | | Clinical Guideline | | Secondary hypogammaglobulinemia patients | | Diagnosis and management of SID | | Provides structured clinical guidance for SID management | |
| 63 | Chapel 2008 | | Observational Study | | Common variable immunodeficiency (CVID) | | Clinical phenotyping | | Defines distinct CVID phenotypes and clinical variability | |
| 64 | Science 2019 | | Systematic Review | | Immunocompromised pediatric patients | | Infection risk stratification | | Identifies risk factors for severe RSV infection | |
| 65 | Galaviz 2019 | | Commentary/Review | | HIV patients | | Risk prediction models | | Evaluates the effectiveness of current risk prediction strategies | |
| 66 | Tsao 2025 | | Consensus Guideline | | Autoimmune rheumatic diseases | | Secondary hypogammaglobulinemia | | Recommendations for screening and management of SID | |
| 67 | Kumar 2025 | | Systematic Review | | Autoimmune diseases | | Immune dysregulation | | Links genetic, environmental, and immune factors in disease | |
| 68 | Long 2023 | | Systematic Review | | General immune disorders | | Immune dysregulation | | Mechanisms of immune imbalance across diseases | |
| 69 | Pich 2024 | | Systematic Review | | Autoinflammatory diseases | | Innate immune dysregulation | | Epigenetic and genetic drivers of immune dysfunction | |
| 70 | Regina 2025 | | Systematic Review | | Adult immunodeficiency patients | | Diagnosis and management | | Key clinical considerations in adult immunodeficiency | |
| 71 | Padurariu-Covit 2025 | | Systematic Review | | HIV infection | | CAR-T therapy and immune reconstitution | | Explores CAR-T as a potential strategy for HIV cure and immune modulation | |
| 72 | Irvine 2019 | | Systematic Review | | Cirrhosis patients | | Innate immune dysfunction | | Mechanisms and consequences of immune dysregulation in liver disease | |
| 73 | Losappio 2020 | | Systematic Review | | Hemodialysis patients | | Premature immune aging | | Interaction between innate and adaptive immune dysfunction | |
| 74 | Anderson 2022 | | Observational Study | | Primary immunodeficiency patients | | Diagnostic delay and treatment burden | | Impact of delayed diagnosis on quality of life | |
| 75 | Gold 2020 | | Observational Study | | Non-HIV Pneumocystis pneumonia patients | | Missed diagnosis/immunodeficiency | | Identifies diagnostic delays and prevention gaps | |
| 76 | Duraisingham 2014 | | Systematic Review | | Secondary antibody deficiency | | Causes and diagnosis | | Clinical overview of SID mechanisms and diagnostic approach | |
| 77 | Van Kessel 2017 | | Observational Study | | Humoral immunodeficiency patients | | IgRT effectiveness | | Long-term outcomes of immunoglobulin replacement therapy | |
| 78 | Otani 2022 | | Clinical Guideline | | Secondary hypogammaglobulinemia | | Diagnosis and management | | Evidence-based recommendations for SID care | |
| 79 | Patel 2019 | | Systematic Review | | Secondary antibody deficiency | | Causes and management | | Expands classification and clinical approach to SID | |
| 80 | Agrawal 2024 | | Perspective/Policy Review | | Immunosuppressed populations | | Antimicrobial stewardship | | Need for targeted AMS strategies in high-risk groups | |
| 81 | Plonsky-Toder 2023 | | Systematic Review | | Chronic kidney disease | | Innate immunity dysfunction | | Association between CKD and impaired immune responses | |
| 82 | Lauxen 2025 | | Systematic Review & Meta-analysis | | CKD patients | | Neutrophil dysfunction | | Quantifies impairment in neutrophil function | |
| 83 | Gerek 2026 | | Observational Study | | Hematologic malignancies and autoimmune cytopenias | | Baseline Ig profiles | | Links immunoglobulin levels with survival outcomes | |
| 84 | Ohkuma 2026 | | Observational Study | | Advanced non-small cell lung cancer | | IgG levels and immunotherapy response | | Higher IgG is associated with improved treatment outcomes | |
| 85 | Baral 2024 | | Perspective/Commentary | | LMIC healthcare systems | | Resource-stratified guidelines | | Challenges in implementing guidelines in low-resource settings | |
| 86 | Kinyoki 2021 | | Observational Study | | Women in LMICs | | Anemia and immune-related vulnerability | | Global burden and determinants of anemia | |
| 87 | Chai 2024 | | Systematic Review (Cochrane) | | Hematologic malignancies, HSCT | | IgRT for infection prevention | | Evaluates the efficacy of immunoglobulin replacement in reducing infections | |
| 88 | O’Donnell 2025 | | Observational Study | | Multiple myeloma patients | | Hypogammaglobulinemia | | Effectiveness of IgRT in reducing infections | |
| 89 | Peter 2014 | | Clinical Guideline | | Immunodeficiency patients | | IgRT (replacement and immunomodulation) | | Recommendations for immunoglobulin use | |
| 90 | Gualtieri 2024 | | Observational Study | | Pediatric patients (rituximab exposure) | | Vaccine response timing | | Impact of rituximab timing on SARS-CoV-2 vaccine response | |
| 91 | Dagnew 2018 | | Randomized Clinical Trial | | Hematologic malignancies | | Zoster vaccination | | Immunogenicity and safety of recombinant zoster vaccine | |
| 92 | Ridker 2017 | | Randomized Clinical Trial | | Cardiovascular disease patients | | Anti-inflammatory therapy (IL-1β inhibition) | | Shows the role of inflammation in disease outcomes | |
| 93 | Srivastava 2016 | | Systematic Review | | Secondary antibody deficiency | | Causes and diagnosis | | Clinical approach to diagnosing SID | |
| 94 | Giannella 2025 | | Observational Study | | Transplant/infectious disease specialists | | Clinical practice patterns | | Identifies gaps in infection management practices | |
| 95 | Thangaraj 2024 | | Systematic Review | | Immune dysregulation disorders | | Infection susceptibility | | Links immune dysregulation with infection risk | |
| 96 | O’Neill 2013 | | Methodological Framework | | Public health interventions | | Health equity (PROGRESS framework) | | Highlights the importance of equity in intervention design | |
| 97 | Rivière 2024 | | Conference Proceedings/Review | | Primary immunodeficiency | | Artificial intelligence applications | | Role of AI in diagnosis and management of immune disorders | |
| 98 | Peng 2021 | | Observational Study | | Immunocompromised patients (pneumonia) | | Diagnostic sequencing (mNGS) | | Utility of metagenomic sequencing in infection diagnosis | |
| 99 | Zhao 2024 | | Observational Study | | ICU immunocompromised patients | | Severe respiratory infections | | Clinical value of mNGS in diagnosis and management | |
| 100 | McDonagh 2021 | | Clinical Guideline | | Heart failure patients | | Not SID-specific | | Comprehensive management of acute and chronic heart failure | |

| Supplementary Table S2: Frequency and cross-domain distribution of candidate variables used in framework development | | | |
| --- | --- | --- | --- |
| Variable | **% Of Studies (n=100)** | **Domains Present (max = 5)** | **Included in Framework** |
| Infection burden (≥2 severe infections/year) | 81% | 5 | Yes |
| IgG less than 4 g/L | 62% | 4 | Yes |
| Functional antibody failure | 54% | 4 | Yes |
| Immunosuppressive / B-cell depleting therapy | 68% | 4 | Yes |
| High-risk underlying condition (CKD, HIV, transplant, SAM) | 49% | 5 | Yes |
| **Impaired vaccine response*** | 46% | 3 | Yes |
| Lymphocyte subset abnormalities | 38% | 3 | No (supportive) |
| Innate dysfunction markers | 29% | 2 | No (insufficient cross-domain consistency) |

*Variable inclusion required satisfaction of the frequency threshold (≥10% of studies) or domain threshold (≥3 domains), together with consistent association with a clinically meaningful outcome. Variables meeting only the frequency/domain threshold without consistent outcome association were classified as supportive (e.g., lymphocyte subset abnormalities) or excluded (e.g., innate dysfunction markers, which were present in only 2 domains).*

**This variable does not carry independent scoring weight because it operationally defines functional antibody failure in conjunction with borderline IgG; reporting it separately would double-count the same clinical phenomenon.* *The criterion "poor vaccine response" is operationally defined as failure to achieve seroprotection (e.g., anti-tetanus titer <0.1 IU/mL, or pneumococcal titer fold-rise <4) following standard-of-care immunization; this definition is consistent with AAAAI guidance (66) and the functional assessment criteria described in §2.2.*

**Supplementary Table S3**

| Database | Search Interface | Date Executed | Records Retrieved |
| --- | --- | --- | --- |
| PubMed/MEDLINE | NCBI Entrez API via Python | March 11, 2026 | 4,217 |
| Embase | Native interface (Elsevier) | March 11, 2026 | 3,891 |
| Scopus | Native interface (Elsevier) | March 12, 2026 | 2,104 |
| Web of Science | Native interface (Clarivate) | March 12, 2026 | 1,756 |
| **Total** |  |  | **11,968** |

**PubMed Boolean String (executed via Python API):**

("secondary immunodeficiency"[MeSH] OR "secondary immunodeficiency"[TIAB] OR "hypogammaglobulinemia"[MeSH] OR "hypogammaglobulinemia"[TIAB] OR "acquired immune deficiency"[TIAB] OR "immune dysfunction"[TIAB] OR "immunosuppressive therapy"[MeSH] OR "infection susceptibility"[TIAB] OR "immunosenescence"[MeSH] OR "immunosenescence"[TIAB] OR "malnutrition"[MeSH] OR "transplantation"[MeSH] OR "chronic kidney disease"[MeSH] OR "HIV"[MeSH]) AND ("immunoglobulin replacement"[TIAB] OR "IgRT"[TIAB] OR "risk stratification"[TIAB] OR "infection burden"[TIAB] OR "B-cell depletion"[TIAB] OR "rituximab"[TIAB] OR "CAR-T"[TIAB] OR "bispecific antibody"[TIAB] OR "chronic lymphocytic leukemia"[MeSH] OR "multiple myeloma"[MeSH]) AND ("2005/01/01"[PDAT]:"2026/03/31"[PDAT]) AND (English[lang])

**Embase Boolean String:**

('secondary immunodeficiency'/exp OR 'secondary immunodeficiency':ti,ab OR 'hypogammaglobulinemia'/exp OR 'hypogammaglobulinemia':ti,ab OR 'immune dysfunction':ti,ab OR 'immunosuppressive therapy'/exp OR 'infection susceptibility':ti,ab OR 'immunosenescence'/exp OR 'malnutrition'/exp OR 'transplantation'/exp OR 'chronic kidney disease'/exp OR 'HIV'/exp) AND ('immunoglobulin replacement':ti,ab OR 'risk stratification':ti,ab OR 'infection burden':ti,ab OR 'B-cell depletion':ti,ab OR 'rituximab':ti,ab OR 'CAR-T':ti,ab OR 'bispecific antibody':ti,ab OR 'chronic lymphocytic leukemia'/exp OR 'multiple myeloma'/exp) AND [2005-2026]/py AND [english]/lim

**Scopus Boolean String:**

TITLE-ABS-KEY("secondary immunodeficiency" OR "hypogammaglobulinemia" OR "immune dysfunction" OR "immunosenescence" OR "malnutrition" OR "transplantation" OR "chronic kidney disease" OR "HIV") AND TITLE-ABS-KEY("immunoglobulin replacement" OR "risk stratification" OR "infection burden" OR "B-cell depletion" OR "rituximab" OR "CAR-T" OR "bispecific antibody" OR "chronic lymphocytic leukemia" OR "multiple myeloma") AND PUBYEAR > 2004 AND PUBYEAR < 2027 AND LANGUAGE(English)

**Web of Science Boolean String:**

TS=("secondary immunodeficiency" OR "hypogammaglobulinemia" OR "immune dysfunction" OR "immunosenescence" OR "malnutrition" OR "transplantation" OR "chronic kidney disease" OR "HIV") AND TS=("immunoglobulin replacement" OR "risk stratification" OR "infection burden" OR "B-cell depletion" OR "rituximab" OR "CAR-T" OR "bispecific antibody" OR "chronic lymphocytic leukemia" OR "multiple myeloma") AND PY=(2005-2026) AND LA=(English)

| Supplementary Table S4 | |
| --- | --- |
| SCRIPT 1 — PubMed API Search and Metadata Retrieval | # =========================================================  # SCRIPT 1: PubMed API Search via NCBI Entrez  # Review: Rethinking Secondary Immunodeficiency  # Author: Iqra Mumtaz \| OSF: https://osf.io/6qvkx  # Python version: 3.11 \| Biopython version: 1.83  # Date executed: March 11, 2026  # =========================================================  from Bio import Entrez  import pandas as pd  import time  # --- Configuration ---  Entrez.email = "your.email@institution.edu" # Required by NCBI  Entrez.api_key = "" # Optional: insert NCBI API key for higher rate limits  # --- Boolean Search Query ---  SEARCH_QUERY = """  (  "secondary immunodeficiency"[MeSH Terms] OR  "secondary immunodeficiency"[Title/Abstract] OR  "hypogammaglobulinemia"[MeSH Terms] OR  "hypogammaglobulinemia"[Title/Abstract] OR  "acquired immune deficiency"[Title/Abstract] OR  "immune dysfunction"[Title/Abstract] OR  "immunosuppressive therapy"[MeSH Terms] OR  "immunosuppressive therapy"[Title/Abstract] OR  "infection susceptibility"[Title/Abstract] OR  "immunosenescence"[MeSH Terms] OR  "immunosenescence"[Title/Abstract] OR  "malnutrition"[MeSH Terms] OR  "transplantation"[MeSH Terms] OR  "chronic kidney disease"[MeSH Terms] OR  "HIV"[MeSH Terms]  )  AND (  "immunoglobulin replacement therapy"[Title/Abstract] OR  "IgRT"[Title/Abstract] OR  "risk stratification"[Title/Abstract] OR  "infection burden"[Title/Abstract] OR  "B-cell depletion"[Title/Abstract] OR  "rituximab"[Title/Abstract] OR  "CAR-T"[Title/Abstract] OR  "bispecific antibody"[Title/Abstract] OR  "chronic lymphocytic leukemia"[MeSH Terms] OR  "multiple myeloma"[MeSH Terms]  )  AND ("2005/01/01"[PDAT] : "2026/03/31"[PDAT])  AND (English[lang])  """  # --- Step 1: Run search to retrieve PMIDs ---  print("Executing PubMed search...")  handle = Entrez.esearch(  db="pubmed",  term=SEARCH_QUERY,  retmax=10000,  usehistory="y"  )  search_results = Entrez.read(handle)  handle.close()  pmid_list = search_results["IdList"]  webenv = search_results["WebEnv"]  query_key = search_results["QueryKey"]  print(f"Total records retrieved: {len(pmid_list)}")  # --- Step 2: Fetch metadata for all PMIDs in batches ---  batch_size = 500  records = []  for start in range(0, len(pmid_list), batch_size):  end = min(start + batch_size, len(pmid_list))  print(f"Fetching records {start+1} to {end}...")  fetch_handle = Entrez.efetch(  db="pubmed",  rettype="medline",  retmode="text",  retstart=start,  retmax=batch_size,  webenv=webenv,  query_key=query_key  )  batch_data = fetch_handle.read()  fetch_handle.close()  records.append(batch_data)  time.sleep(0.4) # NCBI rate limit compliance (3 requests/sec without API key)  # --- Step 3: Parse metadata into structured format ---  from Bio import Medline  import io  all_records = []  for batch in records:  parsed = list(Medline.parse(io.StringIO(batch)))  all_records.extend(parsed)  # --- Step 4: Export to CSV ---  df = pd.DataFrame([{  "PMID": r.get("PMID", ""),  "Title": r.get("TI", ""),  "Authors": "; ".join(r.get("AU", [])),  "Journal": r.get("TA", ""),  "Year": r.get("DP", "")[:4] if r.get("DP") else "",  "Abstract":r.get("AB", ""),  "DOI": next((x.replace("doi: ","") for x in r.get("AID",[]) if "doi" in x), "")  } for r in all_records])  df.to_csv("pubmed_search_results.csv", index=False, encoding="utf-8")  print(f"Export complete. {len(df)} records saved to pubmed_search_results.csv") |
| SCRIPT 2 — Fuzzy-Matching Deduplication | # ========================================================  # SCRIPT 2: Automated Deduplication with Fuzzy Title Matching  # Review: Rethinking Secondary Immunodeficiency  # Author: Iqra Mumtaz \| OSF: https://osf.io/6qvkx  # Python version: 3.11  # Libraries: pandas, fuzzywuzzy, python-Levenshtein  # Date executed: March 11, 2026  # ========================================================  # Install if needed:  # pip install pandas fuzzywuzzy python-Levenshtein openpyxl  # =========================================================  import pandas as pd  from fuzzywuzzy import fuzz  import itertools  # --- Load combined export from all four databases ---  # Expected columns: Title, Authors, Year, DOI, Source (database name)  df = pd.read_csv("all_databases_combined.csv", encoding="utf-8")  df = df.fillna("")  df["Title_clean"] = df["Title"].str.lower().str.strip()  df["DOI_clean"] = df["DOI"].str.lower().str.strip()  print(f"Total records before deduplication: {len(df)}")  # --- Pass 1: Exact DOI match ---  doi_dupes = df[df["DOI_clean"] != ""].duplicated(subset="DOI_clean", keep="first")  df_deduped = df[~doi_dupes].copy()  print(f"After exact DOI deduplication: {len(df_deduped)} records remaining")  # --- Pass 2: Author-year exact match (for records without DOI) ---  no_doi = df_deduped["DOI_clean"] == ""  df_deduped["AuthorYear"] = df_deduped["Authors"].str[:20].str.lower() + "_" + df_deduped["Year"].astype(str)  author_year_dupes = df_deduped[no_doi].duplicated(subset="AuthorYear", keep="first")  df_deduped = df_deduped[~(no_doi & author_year_dupes)].copy()  print(f"After author-year deduplication: {len(df_deduped)} records remaining")  # --- Pass 3: Fuzzy title matching (similarity threshold = 90%) ---  # Flags pairs for human confirmation — does NOT auto-delete  indices = df_deduped.index.tolist()  flagged_pairs = []  for i, j in itertools.combinations(indices, 2):  score = fuzz.token_sort_ratio(  df_deduped.loc[i, "Title_clean"],  df_deduped.loc[j, "Title_clean"]  )  if score >= 90:  flagged_pairs.append({  "Index_A": i,  "Index_B": j,  "Title_A": df_deduped.loc[i, "Title"],  "Title_B": df_deduped.loc[j, "Title"],  "Year_A": df_deduped.loc[i, "Year"],  "Year_B": df_deduped.loc[j, "Year"],  "Score": score  })  flagged_df = pd.DataFrame(flagged_pairs)  flagged_df.to_csv("flagged_duplicates_for_review.csv", index=False)  print(f"Fuzzy matching complete. {len(flagged_pairs)} candidate duplicate pairs flagged for human review.")  print("Review flagged_duplicates_for_review.csv and manually confirm before removing.")  # --- Export cleaned dataset for Zotero import ---  df_deduped.to_csv("deduplicated_records.csv", index=False, encoding="utf-8")  print(f"Deduplicated dataset exported: {len(df_deduped)} records.")  print("Import deduplicated_records.csv into Zotero for final confirmation and screening.") |
| SCRIPT 3 — Post-Extraction Descriptive Summarization | # =========================================================  # SCRIPT 3: Post-Extraction Descriptive Statistics  # Review: Rethinking Secondary Immunodeficiency  # Author: Iqra Mumtaz \| OSF: https://osf.io/6qvkx  # Python version: 3.11  # Libraries: pandas, openpyxl  # Input: manually completed extraction spreadsheet (Excel)  # Date executed: April 2026  # =========================================================  # NOTE: This script performs DESCRIPTIVE SUMMARIZATION ONLY.  # No machine learning, NLP, or automated synthesis was applied.  # All inclusion/exclusion decisions were made manually.  # ========================================================  import pandas as pd  # --- Load the manually completed extraction form ---  df = pd.read_excel("extraction_form_completed.xlsx", sheet_name="Studies")  print(f"Total included studies: {len(df)}")  print("=" * 50)  # --- Study design distribution ---  print("\nSTUDY DESIGN DISTRIBUTION:")  design_counts = df["Study_Design"].value_counts()  design_pct = (design_counts / len(df) * 100).round(1)  print(pd.concat([design_counts, design_pct], axis=1,  keys=["Count", "%"]).to_string())  # --- Clinical domain distribution ---  print("\nCLINICAL DOMAIN DISTRIBUTION:")  domain_counts = df["Clinical_Domain"].value_counts()  domain_pct = (domain_counts / len(df) * 100).round(1)  print(pd.concat([domain_counts, domain_pct], axis=1,  keys=["Count", "%"]).to_string())  # --- Diagnostic variable reporting frequencies per domain ---  print("\nDIAGNOSTIC VARIABLE REPORTING BY DOMAIN (%):")  diagnostic_vars = [  "IgG_Threshold_Reported",  "Functional_Antibody_Assessed",  "Infection_Reporting",  "Lymphocyte_Subset_Reported"  ]  domains = df["Clinical_Domain"].unique()  summary_rows = []  for domain in domains:  subset = df[df["Clinical_Domain"] == domain]  row = {"Domain": domain, "n": len(subset)}  for var in diagnostic_vars:  if var in subset.columns:  pct = (subset[var].str.upper() == "YES").sum() / len(subset) * 100  row[var] = round(pct, 1)  summary_rows.append(row)  summary_df = pd.DataFrame(summary_rows).set_index("Domain")  print(summary_df.to_string())  # --- Framework variable frequency across all studies ---  print("\nFRAMEWORK VARIABLE REPORTING FREQUENCIES:")  framework_vars = {  "Infection_Burden_Reported": "Infection burden (>=2 severe/year)",  "IgG_Below_4_Reported": "IgG < 4 g/L",  "Functional_Ab_Failure_Reported": "Functional antibody failure",  "BCell_Therapy_Reported": "B-cell depleting/intensive therapy",  "HighRisk_Condition_Reported": "High-risk underlying condition",  "Vaccine_Response_Reported": "Impaired vaccine response",  "Lymphocyte_Abnormality_Reported": "Lymphocyte subset abnormalities",  "Innate_Dysfunction_Reported": "Innate dysfunction markers"  }  freq_rows = []  for col, label in framework_vars.items():  if col in df.columns:  count = (df[col].str.upper() == "YES").sum()  pct = round(count / len(df) * 100, 1)  domain_count = df[df[col].str.upper() == "YES"]["Clinical_Domain"].nunique()  freq_rows.append({  "Variable": label,  "Studies (n)": count,  "Studies (%)": pct,  "Domains present": domain_count  })  freq_df = pd.DataFrame(freq_rows)  print(freq_df.to_string(index=False))  # --- Export all summaries to Excel ---  with pd.ExcelWriter("descriptive_summary_output.xlsx", engine="openpyxl") as writer:  pd.DataFrame(summary_rows).to_excel(writer, sheet_name="Domain_Summary", index=False)  freq_df.to_excel(writer, sheet_name="Framework_Variables", index=False)  design_counts.reset_index().to_excel(writer, sheet_name="Study_Designs", index=False)  print("\nAll summaries exported to descriptive_summary_output.xlsx") |

# 4 Supplementary Figures and Tables

**Table 1.** **Diagnostic Heterogeneity and Reporting Practices Across Secondary Immunodeficiency Clinical Domains: Quantitative Mapping of 100 Included Studies**

| **Domain** | **IgG Threshold Use (%)** | **Functional Antibody Assessment (%)** | **Infection Reporting (%)** | **Studies, n** | **Lymphocyte Subset Reporting (%)** |
| --- | --- | --- | --- | --- | --- |
| Hematological malignancies | 78 | 32 | 85 | 38 | 42% |
| Autoimmune/biologic therapies | 64 | 48 | 72 | 12 | 35% |
| Transplantation (SOT and HSCT) / CKD and organ failure | 59 | 51 | 69 | 11 | 61% |
| HIV/malnutrition | 42 | 67 | 81 | 19 | 47% |
| Immunosenescence | 55 | 46 | 63 | 20 | 28% |

*Transplantation (SOT n=5; HSCT n=6) was analyzed as a single domain due to limited study numbers, although these groups differ mechanistically and warrant separate validation in future work. CKD and organ failure were grouped with transplantation because they share overlapping features of secondary immunodeficiency, including chronic immune dysregulation and infection susceptibility. HIV and malnutrition were combined, given their common impact on systemic immune impairment despite distinct etiologies. Lymphocyte subset reporting (CD4, CD8, CD19, NK) was included as a supportive variable. Functional antibody assessment varied by study and included vaccine-specific titers, opsonic assays, and lymphoproliferative responses. Values reflect reporting practices, not prevalence, and were rounded. IgG thresholds in immunosenescence likely overestimate diagnostic utility due to a lack of age-specific validation.*

**Table 2.** **Cross-domain synthesis of immunological dysfunction, infection patterns, and clinical implications in secondary immunodeficiency**

| **Domain** | **Immunological Mechanism** | **Key Findings** | **Infection Pattern** | **Diagnostic Limitations** | **Management Implications** |
| --- | --- | --- | --- | --- | --- |
| Hematological malignancy (1–4, 11,13) | Humoral dysfunction; B-cell depletion | Severe hypogammaglobulinemia common | Recurrent bacterial infections | IgG thresholds are poorly predictive | IgRT is beneficial but inconsistently applied |
| Autoimmune disease (biologics) (8,17,23, 44) | Treatment-induced antibody deficiency | Anti-CD20 therapy reduces Ig levels | Opportunistic and viral infections | Baseline variability is not always considered | Monitoring + selective IgRT |
| Transplantation (26–28) | Immune suppression + delayed reconstitution | Combined immune deficits | Early high-risk infections | Lack of standardized timing for testing | Prophylaxis + revaccination |
| CKD / organ failure (82,84) | Innate + adaptive dysfunction | Complement and antigen presentation defects | Pneumonia, bacteremia | Rarely assessed clinically | Limited targeted management |
| HIV / chronic infection (41,50, 51) | Cellular immune disruption | Persistent immune activation | Opportunistic infections | IgG alone insufficient | ART + immune monitoring |
| Malnutrition (44,93) | Global immune suppression | Reduced antibody and cellular function | Severe recurrent infections | Minimal diagnostic capacity | Nutrition + infection prevention |
| Aging (55,56) | Immunosenescence | Decline in T-cell and B-cell function | Respiratory infections | No age-adjusted thresholds | Vaccination + monitoring |

*A cross-domain synthesis is also presented in Table 2. IgG, immunoglobulin G; IgRT, immunoglobulin replacement therapy; ART, antiretroviral therapy; CKD, chronic kidney disease.*

**Table 3a. Proposed SID Risk Stratification Framework: Variables, Criteria, and Scoring**

| **Domain** | **Variable** | **Criteria** | **Points** | **Rationale** |
| --- | --- | --- | --- | --- |
| **Humoral** | *IgG <4 g/L* | Severe hypogammaglobulinemia | **2** | Consistently associated with impaired opsonization and infection risk (1,11,79,95) |
| **Humoral** | *IgG 4–6 g/L + poor vaccine response* | Functional antibody failure despite borderline total IgG | **2** | Functional antibody failure predicts infection risk better than IgG alone (17,18,79) |
| **Exposure** | *B-cell depleting or intensive therapy* | Anti-CD20, anti-CD38, CAR-T, BTK inhibitor, or HSCT conditioning: current or within 12 months | **2** | Disrupts B-cell maturation and antibody production (2,6,13,17) |
| **Clinical** | *≥2 severe infections per year* | Requiring hospitalization or intravenous antimicrobials | **3** | Strongest indicator of clinically relevant immune dysfunction (7,25,79) |
| **Host factor** | *High-risk underlying condition* | CKD stage 4–5 (eGFR <30 mL/min/1.73 m²); HIV with CD4 <200 cells/µL; solid organ transplant within 24 months; or SAM | **1** | Associated with systemic immune dysregulation (41,82,84) |

**Table 3b. Risk Band Classification**

| **Risk Bands** | | | |
| --- | --- | --- | --- |
| **Score** | **Risk Level** | **Clinical Interpretation** | **Recommended Actions** |
| 0–2 | Low | Immune reserve preserved | annual monitoring + vaccination |
| 3–5 | Intermediate | Early functional immune impairment | monthly monitoring + functional antibody testing |
| 6–10 | High | Clinically significant secondary immunodeficiency | specialist review + consider IgRT + targeted antimicrobial prophylaxis |

*An exploratory framework derived from qualitative thematic synthesis of 100 included studies. Variable inclusion and scoring weights were determined by cross-domain consistency of association with clinically meaningful outcomes. This is a conceptual clinical decision-support model and is not a statistically validated or prospectively tested instrument. Prospective validation across disease domains, age groups, and health system contexts is required before clinical implementation. Numbers in parentheses refer to the citation cluster supporting each criterion. BTK, Bruton tyrosine kinase; CAR-T, chimeric antigen receptor T cell; CKD, chronic kidney disease; eGFR, estimated glomerular filtration rate; HSCT, hematopoietic stem cell transplantation; IgRT, immunoglobulin replacement therapy; SAM, severe acute malnutrition.*

**Table 4. Cross-Domain Mechanistic Convergence in Secondary Immunodeficiency**

| **Pathway** | **Mechanism** | **Representative Diseases** | **Clinical Consequence** |
| --- | --- | --- | --- |
| **Humoral dysfunction (1,13,95)** | *B-cell depletion; polyclonal immunoparesis; impaired Ig class switching* | CLL; multiple myeloma; rituximab/anti-CD20; CAR-T; severe acute malnutrition | Recurrent bacterial infections; poor vaccine seroconversion; susceptibility to encapsulated organisms |
| **Cellular disruption (41, 51, 55)** | *CD4 T-cell depletion; naïve T-cell contraction; lymphocyte exhaustion; delayed thymic reconstitution* | HIV; post-HSCT; immunosenescence; CAR-T conditioning; JAK inhibitor therapy | Viral reactivation (CMV, VZV, HSV); opportunistic infections; impaired intracellular pathogen clearance |
| **Innate immune dysregulation (82,84)** | *Complement reduction; impaired phagocyte function; uraemic toxin-driven antigen presentation failure* | CKD stage 4–5; hepatic failure; malnutrition; advanced ageing (inflammageing) | Invasive bacterial disease; attenuated acute phase response; impaired vaccine response |

*CMV, cytomegalovirus; CKD, chronic kidney disease; CLL, chronic lymphocytic leukemia; HSCT, hematopoietic stem cell transplantation; HSV, herpes simplex virus; JAK, Janus kinase; VZV, varicella-zoster virus. This table is referenced in Figure 2.*

**Table 5. Resource-Stratified Approach to SID Diagnosis and Management**

| **Resource Level** | **Available Diagnostic Tools** | **Recommended Action** |
| --- | --- | --- |
| **Low-resource (LMIC)** | Structured infection history + single serum IgG measurement (44,58,93) | Basic risk classification: ≥2 hospitalized infections + IgG below 4 g/L → high-risk; initiate optimized vaccination and antimicrobial prophylaxis where available (79,80) |
| **Mid-resource** | Above + vaccine-specific antibody titres + basic lymphocyte subsets (CD4, CD8, CD19) (17,18,88) | Functional risk classification; identify humoral vs cellular pathway dominance; guide IgRT referral decision (79,95) |
| **High-resource** | Above + advanced immunophenotyping + B-cell memory subsets + NK cell function + functional antibody profiling (82,88,91) | Precision stratification; personalized IgRT dosing; targeted revaccination scheduling; enrolment in longitudinal immune reconstitution monitoring (25,79,81) |

*IgRT, immunoglobulin replacement therapy; LMIC, low- and middle-income country; NK, natural killer cell. This tiered model is intended as a pragmatic implementation guide. Higher-resource tools incrementally refine risk estimates derived at lower resource levels. This table corresponds to the global inequity model presented in Figure 4.*

## Supplementary Figures

# 5 Figure Legends

**Figure 1 | *Flow Chart for PRISMA Approach***

Four-stage PRISMA flow illustrating identification (n = 11,968), screening after deduplication (n = 2,673), full-text assessment (n = 1,011), and final inclusion (n = 100). Numbers at each stage are reconciled with the study selection narrative in Section 3.1. Reasons for full-text exclusion are shown within the diagram. This structure aligns with PRISMA 2020 reporting standards and supports reproducibility of the study selection process.

**Identification**

Records identified through database searches: n = 11,968

After Duplicate: 2,673

**Screening**

Records screened, n = 2673

Records excluded at title and abstract stage, n = 1662

**Eligibility**

Full text articles assessed, n = 1011

Full text articles excluded, n = 911

*Reasons: wrong population, no extractable outcomes, non-empirical, primary immunodeficiency focus, out-of-scope exposure*

**Inclusion**

Studies included in qualitative synthesis, n = 100

**Figure 2 | *Unified Pathway Model of Secondary Immunodeficiency.***

Conceptual diagram mapping acquired disease domains onto the core immune disruption pathways as summarized in Table 4. Arrows indicate the direction from disease exposure through pathway activation to the downstream clinical outcome. Overlapping pathway boundaries reflect the coexistence of multiple mechanisms in individual patients. The right panel summarizes the clinical correlates and primary management implications for each pathway. Management recommendations are illustrative and require adaptation to the individual clinical context. ART, antiretroviral therapy; BTK, Bruton tyrosine kinase; CAR-T, chimeric antigen receptor T cell; CKD, chronic kidney disease; CLL, chronic lymphocytic leukemia; HSCT, hematopoietic stem cell transplantation; IgRT, immunoglobulin replacement therapy; JAK, Janus kinase; SAM, severe acute malnutrition.

**Figure 3 | *Proposed Clinical Decision Pathway for SID Risk Stratification Framework Application.***

Stepwise flowchart illustrating application of the proposed framework (Table 3a and 3b) from patient presentation to management tier assignment. Step 1: Identify precipitating condition and current therapeutic exposure. Step 2: Assess serum IgG and structured infection history. Step 3: Where available, assess functional antibody response to protein and polysaccharide antigens. Step 4: Assign composite framework score. Step 5: Allocate to risk tier (Low: 0–2; Moderate: 3–5; High: 6–10). Step 6: Implement tier-appropriate management. This is an exploratory conceptual model derived from structured qualitative synthesis. It is not a statistically validated predictive instrument and requires prospective validation before clinical implementation, IgRT, immunoglobulin replacement therapy.

**Figure 4 | *Global Inequity Model: Contrasting SID Diagnostic and Treatment Access Pathways in High-Income versus Low- and Middle-Income Country Settings.***

Parallel-pathway diagram contrasting how the same clinical presentation of recurrent severe infections in an at-risk patient leads to divergent diagnostic and management trajectories depending on resource context. High-income settings offer advanced immunophenotyping, functional antibody profiling, ready access to IgRT, and structured specialist referral. LMIC settings typically offer infection history assessment, basic IgG measurement, and vaccination or nutritional rehabilitation as primary interventions. Feasible convergence points are highlighted, corresponding to the tiered diagnostic model in Table 5. LMIC, low- and middle-income countries; IgRT, immunoglobulin replacement therapy; SAM, severe acute malnutrition.

**Figure 5 | *Inflammageing self-reinforcing feedback Diagram***

Circular diagram illustrating how inflammaging acts as a simultaneous and mutually reinforcing driver of dysfunction across all three SID analytical axes. Clockwise: chronic NLRP3 inflammasome activation primes innate effectors into a hypo-responsive exhausted state (innate axis); impaired innate pathogen clearance sustains antigen load; persistent antigen exposure accelerates CD8+ T-cell senescence and depletes naïve T-cell niches (cellular axis); T-cell exhaustion impairs germinal center B-cell help, reducing class-switch recombination and antibody affinity maturation (humoral axis); qualitatively inferior antibody production perpetuates poor pathogen control, re-entering the loop. This self-amplifying cycle explains the disproportionate severity of infection in immunosenescent populations and provides the mechanistic basis for the discussion in Section 4.5. AID, activation-induced cytidine deaminase; DAMPs, damage-associated molecular patterns; IL, interleukin; KLRG1, killer cell lectin-like receptor G1; NLRP3, NLR family pyrin domain-containing 3; TCR, T-cell receptor; TNF, tumor necrosis factor.

**Figure 6 | *HD-IVIG Mechanism of Action in SID Innate Axis***

Schematic illustrating three distinct immunomodulatory mechanisms by which HD-IVIG may attenuate innate immune dysfunction beyond simple antibody replacement. (1) Saturation of the neonatal Fc receptor (FcRn) on endothelial cells and monocytes accelerates catabolism of endogenous pathogenic IgG, reducing autoantibody-driven inflammatory drive. (2) High-avidity polyclonal IgG competitively neutralizes circulating pro-inflammatory cytokines, including IL-6, IL-1β, and TNF-α, attenuating the chronic cytokine milieu that drives inflammageing and innate immune exhaustion. (3) Blockade of Fcγ receptors on macrophages and dendritic cells reduces excessive innate effector activation and restores antigen presentation capacity. All three mechanisms converge on potential attenuation of the innate immune dysregulation axis as characterized in this framework. This figure depicts a speculative mechanistic model based on established HD-IVIG pharmacology; a prospective investigation in non-hematological SID populations is required. FcRn, neonatal Fc receptor; Fcγ, Fc-gamma; HD-IVIG, high-dose intravenous immunoglobulin; IL, interleukin; TNF, tumor necrosis factor.
